# Collective Mid-Infrared Vibrations in Surface-Enhanced Raman Scattering

**DOI:** 10.1021/acs.nanolett.2c02806

**Published:** 2022-08-29

**Authors:** Niclas S. Mueller, Rakesh Arul, Lukas A. Jakob, Matthew Oliver Blunt, Tamás Földes, Edina Rosta, Jeremy J. Baumberg

**Affiliations:** † NanoPhotonics Centre, Cavendish Laboratory, Department of Physics, University of Cambridge, JJ Thomson Avenue, Cambridge CB3 0HE, United Kingdom; ‡ Department of Physics and Astronomy, University College London, London WC1E 6BT, United Kingdom

**Keywords:** plasmonics, SERS, collective vibration, vibrational exciton, mid-infrared

## Abstract

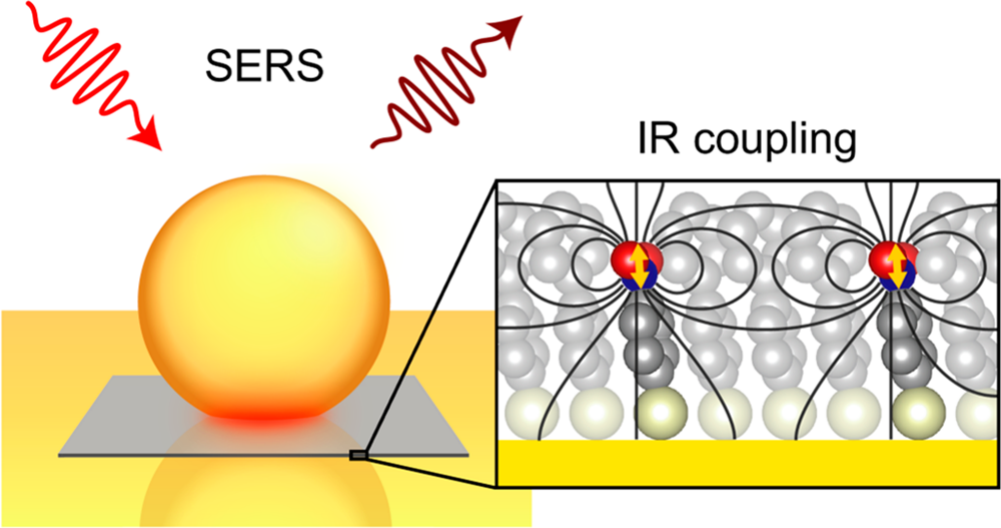

Surface-enhanced Raman scattering (SERS) is typically assumed to occur at individual molecules neglecting intermolecular vibrational coupling. Here, we show instead how collective vibrations from infrared (IR) coupled dipoles are seen in SERS from molecular monolayers. Mixing IR-active molecules with IR-inactive spacer molecules controls the intermolecular separation. Intermolecular coupling leads to vibrational frequency upshifts up to 8 cm^–1^, tuning with the mixing fraction and IR dipole strength, in excellent agreement with microscopic models and density functional theory. These cooperative frequency shifts can be used as a ruler to measure intermolecular distance and disorder with angstrom resolution. We demonstrate this for photochemical reactions of 4-nitrothiophenol, which depletes the number of neighboring IR-active molecules and breaks the collective vibration, enabling direct tracking of the reaction. Collective molecular vibrations reshape SERS spectra and need to be considered in the analysis of vibrational spectra throughout analytical chemistry and sensing.

Vibrational spectroscopy is a powerful tool to probe molecular properties, such as their conformational state, charging, local temperature, and chemical reactions.^1,2^ When molecules are interfaced with metallic nanostructures, their vibrational spectra can be enhanced a billion-fold through surface-enhanced Raman scattering (SERS).^3−5^ Analysis of SERS spectra conventionally assumes that they represent the properties of individual molecules and neglects intermolecular coupling. By contrast, in solid-state materials, vibrations are delocalized across many unit cells, which controls their Raman selection rules.^6,7^ Recently, it was shown that molecules can also be locked into a collective vibration through their optomechanical interaction with a plasmonic nanocavity.^8,9^

While collective vibrations are usually disregarded in Raman, they are well-known in infrared (IR) spectroscopy.^10,11^ In molecular crystals with polar bonds, the coupling of the strong IR transition dipoles (oscillating at IR frequencies *ν*_*vib*_) leads to delocalization of the vibrational wave function as well as a cooperative frequency shift or splitting of the molecular vibrations.^10,12,13^ Because of the similarity to dipole–dipole coupling of Frenkel excitons in molecular crystals, these collective IR vibrations have been historically termed “vibrational excitons” (perhaps confusingly^14^).^10,11^ The cooperative frequency shifts are a powerful tool to monitor crystallization, shape changes, and phase transitions.^15,16^ Because of their sensitivity to the precise intermolecular arrangement, they can track disorder.^12,16^ Such intermolecular coupling is of central importance for electronic transport and photophysics in molecular materials.^12,17^ Recently, signatures of collective IR vibrations were also observed in molecular monolayers on metals, imaged in the near field.^18^ In contrast to optomechanical coupling in SERS, the frequency shifts from collective IR vibrations are independent of incident light intensity, which makes them omnipresent in vibrational spectra. Because of the partially complementary selection rules of IR and Raman spectroscopy and the IR coupling mechanism, collective IR vibrations have rarely been considered in analysis of Raman spectra. However, they can indeed cause major changes in SERS spectra that have implications in its use for diagnostics.

Here, we directly show collective mid-IR vibrations can be detected with SERS. Molecules with polar head groups and strong IR transition dipoles are tightly packed in a self-assembled monolayer (SAM) on a metal surface. Nanoparticle-on-mirror plasmonic cavities are used to record their SERS spectra. By mixing different types of IR-inactive spacer molecules and IR-active molecules, we tune the intermolecular distance and coupling. A collective vibration of the IR-active 4-nitrothiophenol (4NTP) leads to a cooperative frequency shift of 8 cm^–1^ and an asymmetric peak shape in the SERS spectra. We explain both with a microscopic theory of dipole–dipole interactions and compare this quantitatively to density functional theory (DFT) using an extended set of different molecules. Finally, we show how the cooperative frequency shift can monitor photochemical reactions in situ.

We prepare mixed SAMs of the molecules 4NTP and 1,1′-biphenyl-4-thiol (BPT) by coadsorption on atomically flat Au surfaces (SI section S1). 4NTP has a polar NO_2_ headgroup, which when vibrating generates intense electric fields that couple to neighboring molecules (Figure 1a).^13,18^ The vibration is both Raman- and IR-active and therefore ideal to study intermolecular coupling (Figure S3).^18,19^ The molecule BPT acts as a nonpolar spacer to control the intermolecular distance of 4NTP molecules in the mixed SAM. Gold nanoparticles are deposited on the molecular monolayers to form nanoparticle-on-mirror (NPoM) plasmonic cavities.^20^ The NPoM cavities confine light inside the nanometre gap at the metal-molecule interface, enhancing inelastic light scattering by more than 8 orders of magnitude.^21^ This allows SERS to optically read-out the molecular vibrations, probing a few-hundred molecules at each NPoM position on the monolayer.

**Figure 1:**
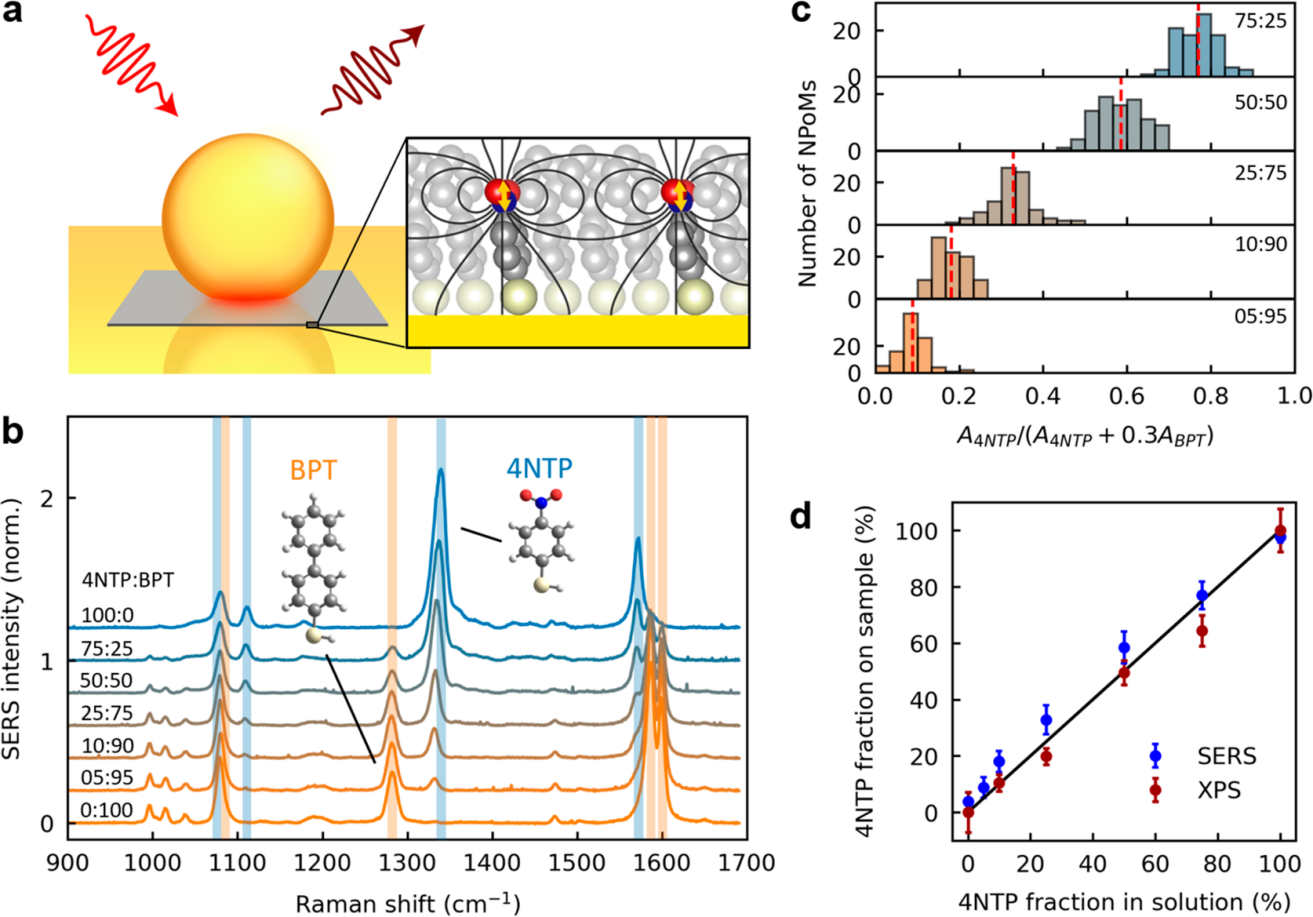
Tuning intermolecular coupling with mixed self-assembled monolayers. (a) Schematic SERS from an NPoM plasmonic cavity, showing incident laser (red), Raman-scattered light (brown), and a molecular monolayer (gray). Inset shows the mixed SAM of the molecules 4NTP and BPT. Polar headgroup vibrations of 4NTP (yellow arrows) generate intense electric fields (black lines) that lead to intermolecular coupling. (b) Average SERS spectra of mixed SAMs of 4NTP and BPT. Labels are mixing fractions in solution during sample preparation. Spectra are normalized by the sum of the 4NTP (1340 cm^–1^, NO_2_ stretch vibration) and BPT (1586 cm^–1^, ring vibration) peak intensities. (c) Statistical analysis from 637 NPoMs of SERS peak ratios of the 1570 cm^–1^ (4NTP, *A*_4NTP_) to the 1586 and 1600 cm^–1^ (BPT, *A*_BPT_) ring vibrations. (d) Mixing fractions of 4NTP and BPT in each sample, from SERS peak ratios (blue, see (c)) and XPS N 1s to S 2p peak ratios (red, see Figure S6). Error bars from statistical variations for SERS and fits for XPS.

To assess the mixing of the two molecules within the SAM, we analyze >7000 SERS spectra from 637 NPoM cavities (SI sections S1 and S6). Average SERS spectra (Figure 1b) for different mixing fractions of the molecules 4NTP (blue) and BPT (orange) show they have complementary vibrations in the spectral range 1000–1600 cm^–1^, including the nitro-stretch of 4NTP at 1340 cm^–1^ and the ring–ring stretch of BPT at 1280 cm^–1^, while their relative intensities tune with mixing fraction. Probing SERS on many NPoM cavities thus measures the local mixing at different positions across a sample (Figures 1c and S10, from ratios of ring vibrations at 1570–1600 cm^–1^). The resulting histograms center at the mixing fractions selected in sample preparation, which confirms effective local mixing and the similar binding affinity of both molecules to Au. Furthermore, the histograms separate well for different mixing fractions with a narrow distribution of peak ratios. This shows that the two molecules mix homogeneously across each sample, allowing us to define *x*_n_ = *A*_4NTP_/(*A*_4NTP_ + 0.3*A*_BPT_) as the fraction of molecules with a nitro group in the mixed SAM, where *A*_4NTP_ and *A*_BPT_ are SERS peak areas (Figure 1c). The scaling factor 0.3 accounts for the different Raman cross sections of the pure SAMs. Given that each NPoM cavity probes only a few hundred molecules on a length scale of ∼10 nm,^22^ we can exclude phase separation into domains of similar molecules, which has been previously observed in some mixed SAMs.^23^

We complement our analysis of SERS peak ratios with X-ray photoelectron spectroscopy (XPS), which measures elemental compositions at metal surfaces (SI section S5).^23−25^ From the sulfur 2p to gold 4f peak ratios we estimate packing densities of 5.0 ± 0.6 nm^–2^ for 4NTP and 4.4 ± 0.5 nm^–2^ for BPT in the pure SAMs (separations *r*_n_ = 4.8 ± 0.3 Å and *r*_BPT_ = 5.1 ± 0.3 Å for hexagonal lattices), in excellent agreement with scanning tunneling microscopy giving *r*_BPT_ = 5.5 ± 0.4 Å (Figure S16).^26^ We measure the molecular mixing from the nitrogen 1s peak coming only from 4NTP and the sulfur 2p peak which comes from all molecules (Figure S6). The XPS peak ratios are in excellent agreement with SERS and match the mixing during sample preparation (Figure 1d). This confirms that SERS peak ratios can be used to probe local mixing in each sample.

The intermolecular vibrational coupling between polar bonds leads to a frequency shift detectable in SERS. The NO_2_ stretch of 4NTP and the ring–ring stretch of BPT are carefully compared for different mixing fractions (Figure 2a). With increasing 4NTP fraction, the NO_2_ vibration shifts to higher wavenumbers and evolves into an asymmetric peak shape. By contrast, the nonpolar BPT vibration remains constant and symmetric. We evaluate this frequency shift by fitting the NO_2_ peak in all individual SERS spectra with an asymmetric Gaussian (different fwhm on either side) to extract the dominant frequency component (Figures 2b and S8). The NO_2_ frequency of 4NTP increases by Δν_NO2_ = 8.1 ± 0.4 cm^–1^ when well-separated molecules in a 5:95 mixed SAM (mean separation *r*_n_ ≃ 2 nm ∝ *x*_n_^–1/2^) are brought into close proximity in the pure SAM (*r*_n_ ≃ 0.5 nm). The BPT frequency instead remains constant within our spectral resolution of 1 cm^–1^ (Figure 2c). The NO_2_ frequency shift scales as *x*_n_^3/2^, which implies that Coulomb dipole–dipole interactions are involved since they scale as *r*_n_^–3^ ∝ *x*_n_^3/2^ (Figure 2b, black line).

**Figure 2:**
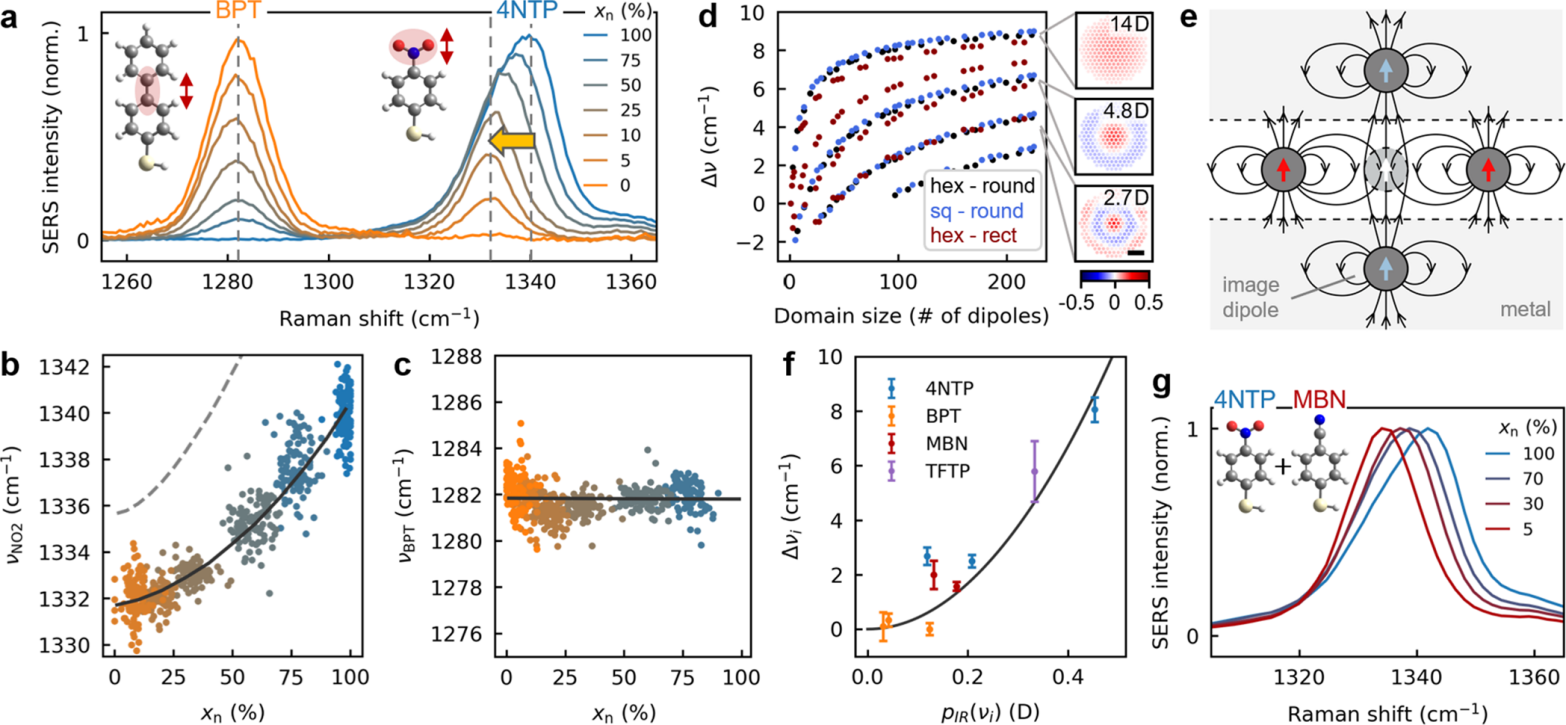
Probing collective mid-IR vibration with surface-enhanced Raman scattering. (a) Average SERS spectra of mixed SAMs of 4NTP and BPT in the region of BPT ring and 4NTP NO_2_ stretch vibrations (insets). (b) Frequencies of the NO_2_ stretch vibration of 4NTP from fits of >7000 individual SERS spectra vs 4NTP fraction *x*_n_. Model predictions of microscopic theory (black line) and when neglecting image dipole coupling (gray dashed). (c) As (b) but for ring–ring stretch vibration of BPT. (d) Cooperative frequency shifts with respect to a single dipole (left) and eigenvectors (right) of the collective modes with the largest net-dipole moment (see labels) for increasing domain sizes, from the microscopic theory of dipole–dipole coupling. Hexagonal (black) and square (blue) lattices inside circular domains and a hexagonal lattice in rectangular domain (aspect ratio 2.5, red) compared, at the same dipole packing density of 4.6 nm^–2^ (vertical dipoles of 0.23 D, *ε*_*m*_ = 2.25). Scale bar is 2 nm. (e) Schematic electric fields of vibrational dipoles (red) and image dipoles (blue) in the plasmonic NPoM gap; see also [^27^]. (f) Experimental frequency shifts Δ*ν*_*i*_ for different vibrations and molecules vs IR transition dipole p_IR_ (*ν*_*i*_) from DFT. (g) Average SERS spectra of the NO_2_ stretch vibration of 4NTP in mixed SAMs with MBN.

The frequency shift can be well-explained using a microscopic theory for the intermolecular coupling of infrared vibrational dipoles **p** (SI section S2).^10,11,18^ These vibrational dipoles are already present without external excitation because of the zero-point vibrations of molecules, which makes their coupling independent of laser intensity (eq S3).^10,11^ The IR dipoles need to be distinguished from the Raman transition dipoles oscillating at optical frequencies *ν*_*L*_ ± *ν*_*vib*_, with *ν*_*L*_ the frequency of the excitation laser. The coupling of molecular vibrations is described by the Hamiltonian
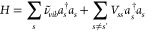
1where *a*_*s*_^†^ and *a*_*s*_ are creation and annihilation operators of molecular vibrations with uncoupled frequencies *ν̃*_*vib*_, while *s* and *s′* are indices for different molecules at lattice sites **r**_*s*_ and **r**_*s*′_. Here
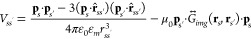
2is the Coulomb interaction potential of two molecules with vibrational dipoles **p**_s_ and **p**_s′_, where *ε*_*m*_ describes the screening by the surrounding molecules. *G⃡*_*img*_ (**r**_*s*_, **r**_*s*′_) is a dyadic Green function that accounts for image dipole coupling through the proximity of molecules to the metallic interfaces (SI section S2).^9^ These image dipoles also renormalize the frequencies of the individual dipoles as *ν̃*_*vib*_ = *ν*_*vib*_ – μ_0_**p**_s_·*G⃡*_*img*_ (**r**_*s*_, **r**_*s*_)·**p**_s_, but this is not detected in our experiments as the interaction remains constant when introducing mixing in the SAM. The collective vibrational frequencies and modes are obtained by calculating the eigenvalues and eigenvectors of the Hamiltonian in eq 1; see SI section S1.

We implement this model for 2D lattices of dipoles with different lattice types and domain shapes. The cooperative frequency upshifts with increasing number of dipoles for the four collective modes with largest net dipoles (Figure 2d). These are little affected by the lattice type and domain shape and mainly depend on the packing density of molecules. The highest frequency mode has the largest net dipole, as all molecules vibrate in-phase. Its frequency rapidly increases with domain size and saturates for >10 × 10 coupled dipoles. The frequency upshift arises from the electric fields of neighboring molecules, which orient oppositely to the vibrating dipole (Figure 2e, red arrows). Higher-order modes have smaller cooperative shifts because subsets of dipoles vibrate out-of-phase, leading to smaller net dipoles (Figure 2d, labels). The proximity of dipoles to the two metal interfaces in the plasmonic gap leads to a series of image dipoles. These generate electric fields that are parallel to the vibrational dipoles and therefore decrease the cooperative frequency up shift (Figure 2e, blue arrows) by 40% for gap sizes of 1 nm (Figure S2). Similarly, a tilt angle ϑ of the molecules decreases the frequency shift by ≃(1 – 1.5sin^2^ϑ). The cooperative frequency shift of the strongest net-dipole mode is
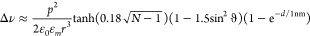
3with IR transition dipole moment *p*, separation *r*, number of coupled dipoles *N*, NPoM gap size *d*, and tilt angle ϑ of the molecules (SI section S2).

The observed frequency shift of the 4NTP NO_2_ vibration is well-explained by a model calculation with *p* = 0.45 D (estimated from DFT), tilt angle ϑ = 34°, and gap size 1 nm as input parameters (Figure 2b black line; parameters: lattice constant *r*_n_ = 0.5 nm·*x*_n_^–1/2^, hexagonal lattice, round domain with 5 nm radius, *ε*_*m*_ = 2.25, dipoles at height 0.7 nm in gap). Ignoring image dipole contributions gives unfeasibly large frequency shifts (gray dashed line). We attribute the peak asymmetry of the NO_2_ vibration to the contribution of higher-order collective modes as well as some molecules that remain uncoupled because of disorder (Figures 2d and S9). The frequency splitting and energetic ordering predicted by our microscopic theory is also well-reproduced by DFT calculations for a tetramer of 4NTP molecules (Figure S4). The DFT also confirms that the highest-frequency collective vibration has the largest Raman activity and that higher-order collective vibrations lead to peak asymmetry. If the collective modes were of similar intensity, the intermolecular coupling would lead to a peak broadening instead of the frequency shift observed experimentally.

The cooperative frequency shift is a general phenomenon for infrared-active molecular vibrations and not specific to the molecule 4NTP. We characterize the vibrational frequency shifts of four different molecules that are diluted in mixed SAMs and compare them to the IR transition dipole *p* from DFT (Figures 2f and S11–S13). The frequency shifts follow the *p*^2^ scaling predicted by eq 3, with the NO_2_ vibration of 4NTP having the largest shift of 8 cm^–1^ (*p* = 0.45 D). Another molecule with strong shifts is 4-(trifluoromethyl)-thiophenol (TFTP) whose polar CF_3_ vibration (*p* = 0.33 D, *v*_vib_ = 1330 cm^–1^) downshifts by 5.8 ± 1.1 cm^–1^ when diluting the molecule in BPT, revealing similar peak asymmetries as 4NTP in the pure SAM (Figure 2f, purple, and Figure S12). Note no correlation is observed between frequency shifts and their SERS intensities (Figure S13e). This is expected as the induced Raman dipoles remain below 0.01 D for the continuous-wave laser powers of 10 μW used here (SI section S3). Illumination with a pulsed laser would be needed to observe any frequency shift from the coupling of induced Raman dipoles.^9^

To exclude other possible intermolecular coupling mechanisms, we diluted 4NTP with the molecule 4-mercaptobenzonitrile (MBN) in mixed SAMs. The NO_2_ headgroup of 4NTP has a static dipole of ∼4 D, which generates static electric fields at the positions of neighboring molecules, in addition to the oscillating fields. This could lead to shifts of molecular vibrations through the vibrational Stark effect.^28^ The CN headgroup of MBN has a similar static dipole as 4NTP and static dipole–dipole interactions should thus not change in mixed SAMs of MBN with 4NTP. However, we observe the same frequency shift 8.6 ± 1.5 cm^–1^ of the NO_2_ vibration as in a mixed SAM with BPT (Figure 2g), implying that IR dipole coupling is instead the cause. Hydrogen bonding and other chemical interactions with neighboring molecules play a negligible role as the same frequency shift is measured irrespective of which molecule is mixed with 4NTP. Our experiments concur with prior work by Gray et al. using IR near-field microscopy, examining mixed SAMs of 4NTP and benzenethiol but only in the IR and without any visible lasers.^18^ Here, we advance this concept of collective IR vibrations considerably by showing its general applicability in SERS for different chemical groups while providing a statistical analysis confirming full molecular mixing. We also develop a detailed theory incorporating quantitative comparisons to DFT. By analyzing overall more than 10,000 SERS spectra from mixtures of four different molecules, we thus provide a comprehensive picture of all possible interactions.

Finally, we demonstrate an application of the cooperative frequency shift to monitor a photochemical reaction at the metal-molecule interface (Figure 3). The molecule 4NTP is well-known for its photocatalytic reduction at metal surfaces, which is relevant for industrial production of aniline and water pollution treatments.^29−33^ When illuminated with high laser intensity, the molecules dimerize to 4,4′-dimercaptoazobenzene (DMAB, Figure 3c).^29−31^ DMAB is a long-lived reaction intermediate, which may be further reduced to 4-aminothiophenol (4ATP). As both 4NTP and DMAB have intense Raman vibrations, the photochemical reaction can be monitored with SERS.^30^ Over time, the SERS spectra evolve here as the laser power is stepwise increased (Figure 3a,b). New SERS lines appear from the N–N (1390 and 1435 cm^–1^) and C–N (1140 cm^–1^) vibrations of DMAB (Figure 3a, red). The SERS intensities of 4NTP lines on the other hand decrease (Figure 3a, blue, 3b). Most strikingly, the NO_2_ vibration of 4NTP shifts irreversibly to lower wavenumbers during the photochemical reaction (Figure 3d). The frequency shift of this line correlates with the decrease in SERS intensity of 4NTP and simultaneous increase in DMAB intensity (Figure 3e).

**Figure 3:**
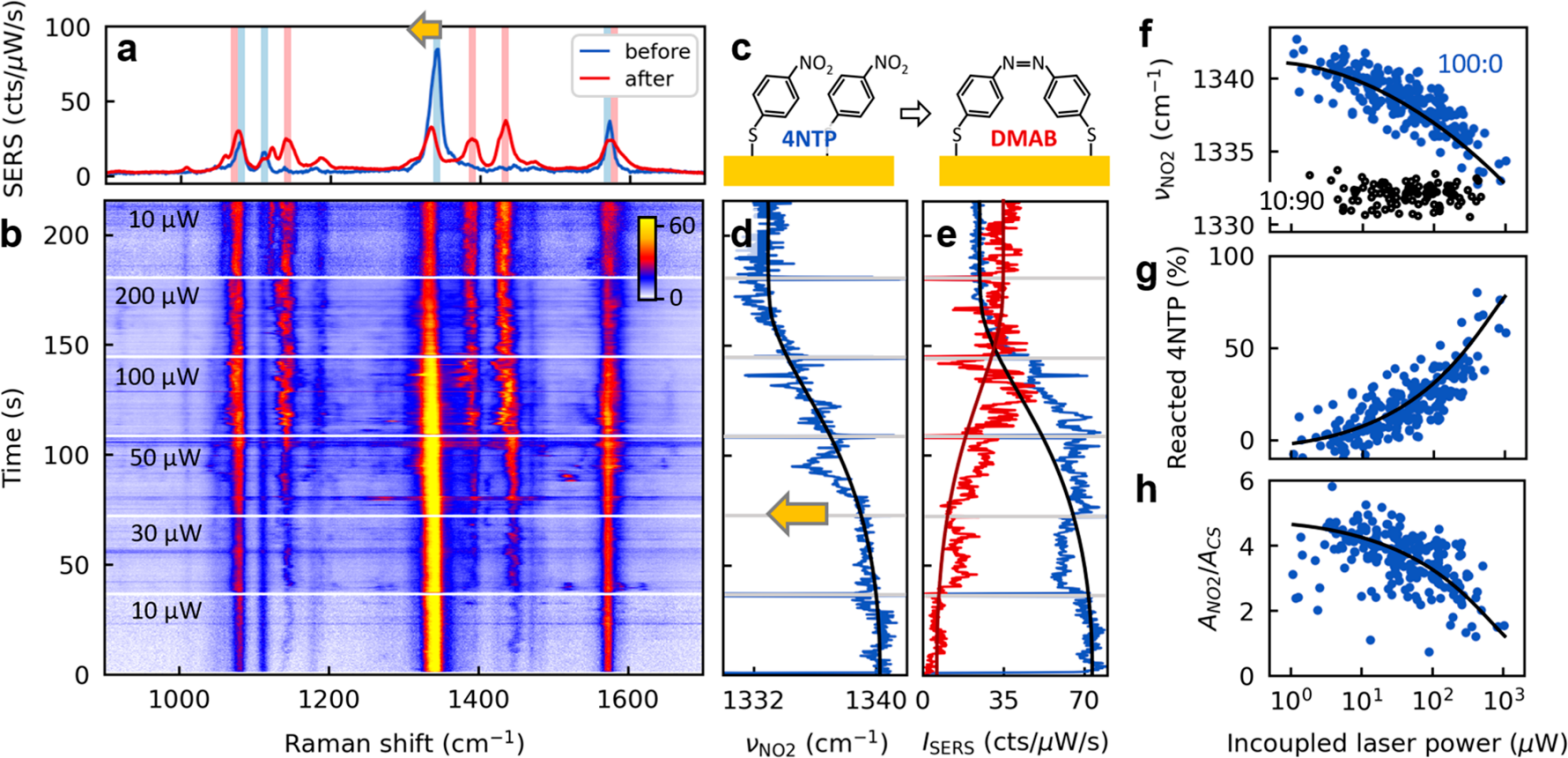
Monitoring photochemical reactions through collective vibrations. (a) SERS spectra of 4NTP before (blue) and after (red) photochemical reaction to DMAB, laser power 10 μW. Main SERS vibrations of the two molecules are highlighted in blue for 4NTP and red for DMAB. (b) SERS time trace of 4NTP while stepwise increasing the laser power (see labels). Color scale bar in cts/μW/s. Time-averaged spectra at 10 μW are shown in (a). (c) Sketch of the photocatalytic dimerization reaction of 4NTP to DMAB; see also [^31^]. (d) Frequency ν_NO2_ and (e) SERS intensity of the 4NTP NO_2_ vibration (blue) from time trace in (b); SERS intensity of DMAB (1435 cm^–1^) in red. Line in (d) is a guide to the eye, used for expected intensity changes in (e). (f) Statistical analysis of ν_NO2_ vs incoupled laser power (see main text) for a pure SAM of 4NTP (blue) and a 10:90 4NTP:BPT mixed SAM (black). (g) Fraction of photoreacted 4NTP molecules, estimated from frequency shifts in (f) and Figure 2b. (h) SERS peak ratio of NO_2_ vibration (only from 4NTP) and ring-sulfur vibration (1080 cm^–1^, all molecules) to quantify fraction of 4NTP molecules. Lines are guides to the eye.

To better understand this, we analyze frequency shifts in laser-power-dependent SERS spectra of more than 70 NPoM cavities (Figures 3f, S14, and S15). The local light intensity at the metal-molecule interface depends on the incident laser power as well as the incoupling efficiency and local field enhancement of each individual NPoM. A plasmonic cavity with stronger incoupling will cause a faster photochemical reaction. We therefore estimate the incoupled light intensity from the SERS intensity at lowest laser power,^9^ referencing the frequency shifts to incoupled power *P*_*i*_ = *P*_*L*_·*I*_*SERS*_^10 μW^/⟨*I*_*SERS*_^10 μW^⟩, for incident laser power *P*_*L*_, collected SERS intensity *I*_*SERS*_^10 μW^ of individual NPoMs at *P*_*L*_ = 10 μW, and average SERS intensity ⟨*I*_*SERS*_^10 μW^⟩ of all NPoMs at this power. At higher laser powers, the NO_2_ frequency in a pure SAM of 4NTP drops from the collective state at 1340 cm^–1^ toward the uncoupled state at 1331.5 cm^–1^ (Figure 3f, blue, and Figure S14; compare to Figure 2b). This is expected as the photochemical reaction requires neighboring NO_2_ groups, and once they react, they no longer participate in the collective vibration. In the strongly diluted 10:90 mixed SAM of 4NTP and BPT, the frequency remains constant (at the uncoupled state), irrespective of laser power (Figure 3f, black, Figure S15) because 4NTP molecules are sufficiently spaced to prevent dimerization and are already uncoupled. Indeed, we observe an increase of DMAB intensity with laser power in the SERS spectra of the pure SAM, while no DMAB peaks are detected for the mixed SAM (Figures S14 and S15). Photochemical charging is implausible to account for the peak shifts here, as we observe no shifting in the mixed SAM (where anions would still be expected) and no additional peaks specific to 4NTP anions.^34,35^

From the frequency shifts during photoreaction (Figure 3f) compared to mixed SAMs (Figure 2b), the fraction of photoreacted 4NTP molecules can be estimated (Figure 3g). With pure 4NTP SAMs, this increases nonlinearly with laser power up to 40–50% for most NPoM cavities. This implies that only half of the 4NTP molecules dimerize, which is below the expected saturation coverage of ∼90% when randomly filling a 2D lattice with dimers.^36^ We attribute this to possible steric and kinetic trapping of the 4NTP molecules that prevents twisting or flexing. Previous studies relied exclusively on SERS intensities to quantify the fraction of photoreacted molecules, but these are prone to fluctuations and drift, especially at high laser powers (Figure 3b,e). For quantitation, the intensity ratio of the NO_2_ vibration (4NTP only) to the ring-sulfur vibration (all molecules) is extracted (Figure 3h). This decreases by ∼40%, which nicely matches the estimated fraction of photoreacted molecules. The SERS intensity ratios are as expected noisier (Δ_RMS_ = 15%) than the frequency shifts (Δ_RMS_ = 9%, compare Figure 3g,h). The cooperative frequency shift of collective molecular vibrations is thus a complementary and preferred tool to monitor photochemical reactions at such metal-molecule interfaces.

In summary, we show that collective vibrations in molecular monolayers lead to frequency shifts detectable by SERS. Analyzing >10,000 SERS spectra from mixtures of different molecules reveals that collective vibrations are a widespread phenomenon among molecules with IR-active vibrations. The cooperative frequency shifts are particularly large for aromatic molecules with polar head groups, such as NO_2_ or CF_3_, and scale with their IR dipole strength. The frequency shifts are well-explained by a microscopic model of vibrational dipole–dipole coupling and DFT.

While collective IR vibrations have been widely studied with IR spectroscopy, they are commonly neglected in SERS. Our work shows that the cooperative frequency shifts from collective vibrations must be included in the analysis of SERS spectra, with implications for analytical chemistry and sensing applications. The cooperative frequency shift can be used as a ruler to measure the intermolecular distance and disorder in situ, as we demonstrate for a photochemical reaction at the metal-molecule interface. This opens the way to measure angstrom scale changes in intermolecular separation with far-field techniques. Collective vibrations can potentially lead to coherent chemical reactions, change energy transport in molecular monolayers, decrease thresholds for vibrational nonlinearities and enhance molecular frequency upconversion of mid-IR light,^8,17,22^ suggesting many future experiments.

## Notes

Source data can be found at: https://doi.org/10.17863/CAM.87880.

## Supporting Information Available

Experimental and theoretical methods; microscopic theory; IR and Raman dipoles from DFT; DFT simulations; characterization of mixed SAMs with XPS; cooperative frequency shifts in mixed SAMs of different types of molecules; monitoring photochemical reactions with SERS; scanning tunneling microscopy of SAM (PDF)
