# Supplementary material for: Collective Mid-Infrared Vibrations in Surface-Enhanced Raman Scattering
Source: Nano Lett. 2022 Aug 29;22(17):7254–60. doi: 10.1021/acs.nanolett.2c02806 (PMC9479150; doi:10.1021/acs.nanolett.2c02806)
Supplement: Supplementary file 1 — nl2c02806_si_001.pdf [file nl2c02806_si_001.pdf]

# Supplementary Information

## Collective Mid-Infrared Vibrations in Surface-Enhanced Raman Scattering

Niclas S. Mueller[1], Rakesh Arul[1], Lukas A. Jakob[1], Matthew Oliver Blunt[2], Tamás Földes[2], Edina Rosta[2], Jeremy J. Baumberg[1*]

[1] NanoPhotonics Centre, Cavendish Laboratory, Department of Physics, JJ Thompson Avenue, University of Cambridge, Cambridge, CB3 0HE, United Kingdom

[2] Department of Physics and Astronomy, University College London, London WC1E 6BT, United Kingdom

[*]email: jjb12@cam.ac.uk

**Index to contents of Supplementary Information:**

Section S1: Experimental and theoretical methods
Section S2: Microscopic theory of collective MIR vibrations in NPoM cavity
Section S3: IR transition dipole moments and induced Raman dipole moments from DFT
Section S4: DFT simulations
Section S5: Characterization of mixed SAMs with X-ray photoelectron spectroscopy
Section S6: Cooperative frequency shifts in mixed SAMs of different types of molecules
Section S7: Monitoring photochemical reactions with SERS
Section S8: Scanning tunnelling microscopy of molecular monolayer

## Section S1: Experimental and theoretical methods

**Sample synthesis.** Mixed self-assembled monolayers of aromatic thiols were prepared by co-adsorption on a template-stripped Au surface. We prepared 1 mM solutions of the molecules 4-nitrothiophenol (4NTP), 4-(trifluoromethyl)-thiophenol (TFTP), 4-mercaptobenzonitrile (MBN), and biphenyl-4-thiol (BPT) in 200 proof anhydrous ethanol. The molecular solutions were then mixed with different molar mixing fractions. All chemicals were purchased from Sigma Aldrich at the highest purity available and used as received. Template-stripped Au samples (100 nm Au on glass, UV glue Norland 81 from Thorlabs) were placed in the solutions overnight excluding ambient light. The samples were rinsed with ethanol and blow dried with nitrogen. 80 nm Au nanoparticles (BBI Solutions) were mixed with 0.1M $NaNO_3$ (10:1), drop casted onto the samples and washed off with DI water after 10 s to form plasmonic NPoM cavities. For the XPS measurements we used thermally evaporated 5 nm Cr + 100 nm Au on Si as substrates.

**Surface-enhanced Raman spectroscopy.** Raman spectra were recorded with a fully automated custom-built setup, consisting of a dark field microscope with motorized stages, as well as spectrometers. The light of a 633 nm CW laser was focused onto the sample with a 0.9 NA 100x objective. The laser power was kept at 10 µW unless stated otherwise. We used dark field (DF) illumination (Olympus BX51 illuminator arm) with an incoherent white light source to locate NPoM cavities through their scattered light. The laser was then automatically centred on each NPoM cavity. The Raman-scattered light was detected with the same objective and guided to a Shamrock spectrometer (SR-303i-B) with an iDus CCD camera (Andor DU416A). We used a 1200 gr/mm grating with 1.2 $cm^{-1}$ resolution for the statistical analysis of mixed SAMs and a 600 gr/mm grating with 2.8 $cm^{-1}$ resolution for recording time traces of the photochemical reaction. Before each measurement, the spectrometer was calibrated with a neon lamp. We also recorded the DF scattering spectra of each NPoM cavity with a fibre-coupled QE Pro spectrometer (Ocean Optics). Using particle tracking algorithms, we recorded the time-dependent SERS spectra and DF spectra of $\sim$100 NPoM cavities on each sample. All spectra were background subtracted using spectra recorded on mirror Au and corrected for the spectral efficiency of the spectrometer. All time-dependent SERS spectra were pre-screened and spectra with transient SERS lines discarded from further analysis. Average spectra were calculated from the normalized individual spectra, and multiplied by the average SERS counts of all spectra. The SERS from 4NTP is particularly strong because of an electronic resonance close to our laser excitation wavelength of 633 nm.[1]

**X-Ray photoelectron spectroscopy.** We characterized the mixed SAMs on a Au surface with a ThermoFisher Escalab 250Xi XPS spectrometer using a monochromatic Al Kα X-ray source. Survey spectra were recorded with a pass energy of 100 eV and high-resolution spectra of elemental peaks with 50 eV pass energy. We refer to Section S5 for more details. The XPS spectra confirm that samples are free of surface contamination and that all molecules are bound to the Au surface (Fig. S5).

**Scanning tunnelling microscopy.** We characterized SAMs of BPT with scanning tunnelling microscopy (STM) using a Keysight 5500 system. STM tips were mechanically cut from a 0.2 nm diameter PtIr (90:10) wire. Multiple tips were used to image each sample to minimise the possibility of tip-induced artefacts influencing the results. Imaging was performed in constant current mode with set-point tunnel currents of between $I_t = 0.2 - 0.5$ nA and sample bias voltages between $V_t = 0.2 - 0.4$ V. Further details are described in Section S8.

**Microscopic theory.** We modelled the cooperative frequency shift of collective mid-IR vibrations with a microscopic theory of Coulomb dipole-dipole interactions. This approach is historically known as the 'vibrational exciton model'.[2] To calculate the intermolecular coupling in a self-assembled monolayer of molecules, we modelled the molecular vibrations with uncoupled frequency $\nu_{vib}$ as point dipoles

**p**. The point dipoles were arranged in 2D lattices, with parallel orientation. Following Refs. 3, 4, we write the Hamiltonian in Eq. (1) as a matrix

$$\boldsymbol{\mathcal{H}} = \begin{pmatrix} \tilde{\nu}_{vib} & V_{1,2} & V_{1,3} & & \\ V_{2,1} & \tilde{\nu}_{vib} & V_{2,3} & \cdots & V_{2,N} \\ V_{3,1} & V_{3,2} & \tilde{\nu}_{vib} & & \\ & \vdots & & \ddots & \vdots \\ & V_{N,2} & & \cdots & \tilde{\nu}_{vib} \end{pmatrix}, \quad \text{(S1)}$$

for a lattice of $N$ dipoles. The cooperative frequencies were obtained from the eigenvalues of $\boldsymbol{\mathcal{H}}$ as

$$\Delta\nu = \mathrm{eig}(\boldsymbol{\mathcal{H}} - \boldsymbol{I}\,\tilde{\nu}_{vib}), \quad \text{(S2)}$$

where $\boldsymbol{I}$ is the identity matrix. The collective vibrational modes were calculated from the eigenvectors of $\boldsymbol{\mathcal{H}}$. We accounted for a tilt angle of the molecules, as well as image dipoles at the metal interfaces of the nanometre gap of the plasmonic NPoM cavities. We refer to Section S2 for more details.

**Density functional theory calculations.** DFT calculations were carried out with the software package Gaussian 16 using the hybrid exchange-correlation functional B3LYP. For C, H, N, O, F, and S atoms we used the basis set 6-31+G(d,p), and for Au atoms the basis set LanL2DZ. We calculated the IR intensity and Raman activity of 4NTP, MBN, TFTP and BPT molecules bound to a single Au atom, after geometry optimization (Fig. S3). The vibrational dipole moment $p_k^{IR}$ was estimated from the DFT IR intensity $I_{IR,k}$ of a vibration with frequency $\nu_k$ as

$$p_k^{IR} = \sqrt{\frac{\hbar I_{IR,k}}{4\pi\nu_k}}, \quad \text{(S3)}$$

where $I_{IR,k}$ is in units of $D^2$ $Å^{-2}$ $amu^{-1}$; see Section S3 for details. Furthermore, we calculated the collective vibrations of a gold-attached 4NTP tetramer with the same functional with additional GD3BJ dispersion correction and the Def2-SVP basis set, see Section S4.

## Section S2: Microscopic theory of collective MIR vibrations in NPoM cavity

We consider a lattice of parallel point dipoles $\mathbf{p}$ that are arranged in a 2D lattice. In our experiments, the molecular monolayer is inside the nanometre gap of a plasmonic NPoM cavity. The metallic interfaces modify the intermolecular coupling through image dipole interactions.[5] We account for this image dipole coupling through a dyadic Green function $\overleftrightarrow{G}_{img}(\mathbf{r}_s, \mathbf{r}_{s'})$, see Eq. (2) in main text, which we derive in the following. We assume that the dipoles are positioned at height $z_0$ between two infinite metallic plates with interfaces at $z = \pm\frac{d}{2}$, where $d$ is the NPoM gap size (Fig. S1). A dipole $\mathbf{p} = (p_x, p_y, p_z)$ at position $\mathbf{r}_0 = (0,0,z_0)$ generates two image dipoles $\mathbf{p}_{img,1} = \beta(-p_x, -p_y, p_z)$ at $\mathbf{r}_{img,1,\pm} = (0,0,\pm d - z_0)$, where $+$ refers to the top, and $-$ to the bottom metal interface. The relative strength of the image dipole is given by the Fresnel reflection coefficient

$$\beta = \left|\frac{\sqrt{\varepsilon_{Au}} - \sqrt{\varepsilon_m}}{\sqrt{\varepsilon_{Au}} + \sqrt{\varepsilon_m}}\right|^2, \tag{S4}$$

where $\varepsilon_{Au}$ is the permittivity of Au and $\varepsilon_m$ the background permittivity of the molecular SAM in the NPoM gap. At mid-IR frequencies Au is an almost perfect reflector with $\varepsilon_{Au} \approx -2900 + 900i$ while $\varepsilon_m$ remains small, such that $\beta \approx 0.98$.[6] The two image dipoles generate another set of image dipoles $\mathbf{p}_{img,2} = \beta^2(p_x, p_y, p_z)$ at $\mathbf{r}_{img,2,\pm} = (0,0,\pm 2d + z_0)$, and so forth. This leads to a lattice of image dipoles

$$\mathbf{p}_{img,j} = \beta^j\left((-1)^j p_x, (-1)^j p_y, p_z\right) \tag{S5}$$

at positions

$$\mathbf{r}_{img,j,\pm} = \left(0,0,\pm jd + (-1)^j z_0\right), \tag{S6}$$

with $j = 1,2,3,\ldots,N_{img}$.

Each image dipole generates an electric field

$$\mathbf{E}_{img,j,\pm}(\mathbf{r}_s) = \frac{3\left(\mathbf{p}_{img,j} \cdot \hat{\mathbf{R}}_{sj,\pm}\right)\hat{\mathbf{R}}_{sj,\pm} - \mathbf{p}_{img,j}}{4\pi\varepsilon_0\varepsilon_m R_{sj\pm}^3} \tag{S7}$$

at lattice site $\mathbf{r}_s$ in the molecular monolayer, where $\mathbf{R}_{sj,\pm} = (\mathbf{r}_{img,j,\pm} - \mathbf{r}_s)/\left|\mathbf{r}_{img,j,\pm} - \mathbf{r}_s\right|$ and $R_{sj,\pm} = \left|\mathbf{R}_{sj,\pm}\right|$. The total electric field that is generated at the lattice position $\mathbf{r}_s$ by all image dipoles from a dipole at $\mathbf{r}_0$ is

$$\mathbf{E}_{img}(\mathbf{r}_s, \mathbf{r}_0) = \mu_0 \overleftrightarrow{G}_{img}(\mathbf{r}_s, \mathbf{r}_0) \cdot \mathbf{p}, \tag{S8}$$

with

$$\overleftrightarrow{G}_{img}(\mathbf{r}_s, \mathbf{r}_{s'}) \cdot \mathbf{p} = \frac{1}{\mu_0}\sum_{j=1}^{N_{img}} \left\{\mathbf{E}_{img,j,+}(\mathbf{r}_s - \mathbf{r}_{s'}) + \mathbf{E}_{img,j,-}(\mathbf{r}_s - \mathbf{r}_{s'})\right\} \tag{S9}$$

where $\overleftrightarrow{G}_{img}(\mathbf{r}_s, \mathbf{r}_{s'})$ is the dyadic Green function that describes the electric field from all image dipoles.

The image dipoles contribute to the Coulomb interaction potential via

$$V_{ss'}^{img} = -\mathbf{p} \cdot \mathbf{E}_{img}(\mathbf{r}_s, \mathbf{r}_{s'}) = -\mu_0 \mathbf{p} \cdot \overleftrightarrow{G}_{img}(\mathbf{r}_s, \mathbf{r}_{s'}) \cdot \mathbf{p}, \tag{S10}$$

which leads to an additional frequency shift of the molecular vibrations. This also includes the self-interaction of a dipole with its image dipoles, which modifies the individual frequencies as

$$\tilde{\nu}_{vib} = \nu_{vib} - \mu_0 \mathbf{p} \cdot \overleftrightarrow{G}_{img}(\mathbf{r}_s, \mathbf{r}_s) \cdot \mathbf{p}. \tag{S11}$$

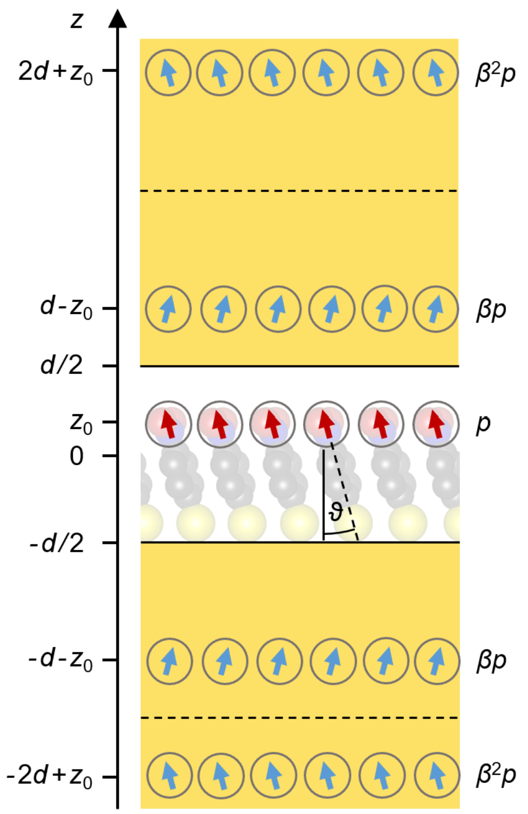


**Figure S1.** Sketch of image dipole lattice (blue) generated by dipoles $p$ (red) that are positioned at height $z_0$ between two metallic plates with gap size $d$. Dipoles have tilt angle $\vartheta$ with respect to $z$ axis.

We implement the microscopic theory for a hexagonal lattice of dipoles and study its dependence on dipole number $N$, tilt angle $\vartheta$, and gap size $d$ (Fig. S2). We find the following dependencies, from which we derive Eq. (3) in the main text:

$$\Delta\nu(N) \approx \Delta\nu(\mathrm{N} \to \infty) \cdot \tanh\left(0.18\sqrt{N-1}\right) \tag{S12}$$

$$\Delta\nu(\vartheta) \approx \Delta\nu(\vartheta = 0) \cdot (1 - 1.5\sin^2\vartheta) \tag{S13}$$

$$\Delta\nu(d) \approx \Delta\nu(d \to \infty) \cdot \left(1 - \mathrm{e}^{-d/1\mathrm{nm}}\right) \tag{S14}$$

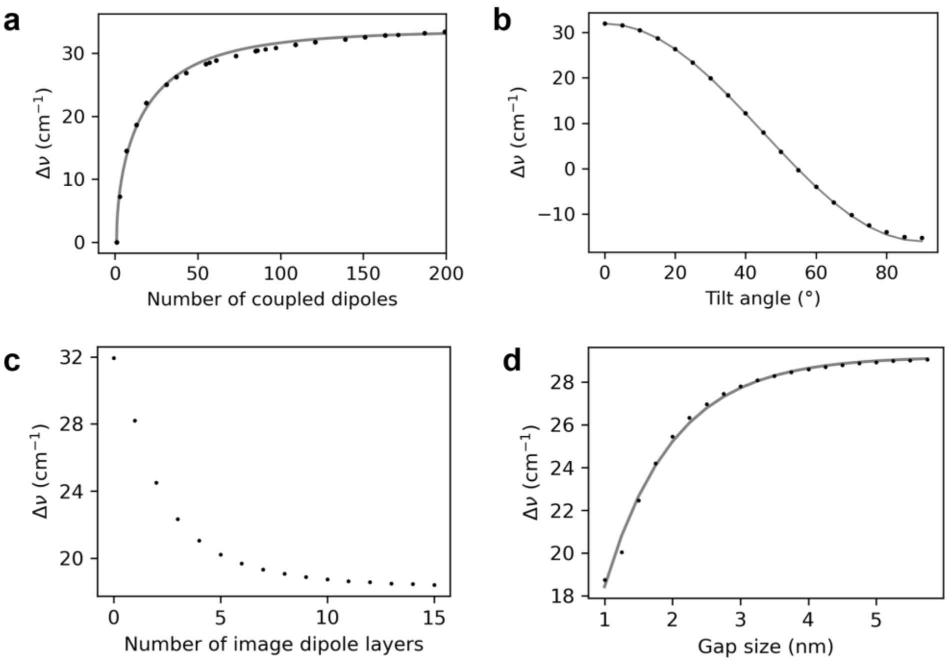


**Figure S2.** Model calculations of cooperative frequency shifts $\Delta\nu$ with microscopic theory. (a) Dependence of $\Delta\nu$ on number of coupled dipoles. Grey line is fit with Eq. (S12). (b) Dependence of $\Delta\nu$ on tilt angle with respect to the $z$ axis. Grey line is fit with Eq. (S13). (c) Dependence of $\Delta\nu$ on number of image dipole layers that are considered, for an NPoM gap size of 1 nm and dipoles positioned at $z_0$ = 0.2 nm. The frequency shift converges for more than 10 layers. (d) Dependence of $\Delta\nu$ on NPoM gap size, with dipoles positioned 0.7 nm above the bottom metal interface. 10 image dipole layers are considered. Grey line is fit with Eq. (S14). All calculations are for hexagonal lattices with round domain shapes, 0.5 nm lattice constant, $\varepsilon_m$ = 2.25, $p$ = 0.45 D.

## Section S3: IR transition dipole moments and induced Raman dipole moments from DFT

The IR transition dipole for a vibrational transition $|0_k\rangle \rightarrow |1_k\rangle$ with frequency $\nu_k$ is given by[7]

$$p_{k,\alpha}^{IR} = \left(\frac{\partial p_\alpha}{\partial Q_k}\right)_0 \langle 1_k|Q_k|0_k\rangle = b_k \left(\frac{\partial p_\alpha}{\partial Q_k}\right)_0, \tag{S15}$$

where $Q_k$ is the normal mode coordinate of the vibration and $p_\alpha$ the cartesian component $\alpha$ of the dipole moment of the molecule. The derivatives are calculated for the minimized structure, indicated by the index 0. Here $b_k = \sqrt{\hbar/4\pi\nu_k}$ is the zero-point amplitude of the vibration. Gaussian DFT calculations provide the IR intensity of molecular vibrations as

$$I_{IR,k} = \sum_\alpha \left(\frac{\partial p_\alpha}{\partial Q_k}\right)_0^2. \tag{S16}$$

When choosing a coordinate system along the vibrational coordinate $k$, we obtain Eq. (S3). We used the conversion factor $\frac{\mathrm{D}^2\ \text{Å}^{-2}\mathrm{amu}^{-1}}{42.26561\ \mathrm{km\ mol}^{-1}}$ to convert the DFT IR intensity from km/mol to $\mathrm{D}^2\ \text{Å}^{-2}\mathrm{amu}^{-1}$.

The induced Raman dipoles, oscillating at the visible frequency $\nu_L - \nu_k$, are given by

$$\mathbf{p}_k^{Raman} = \overleftrightarrow{\alpha}_k \cdot \mathbf{E}_L, \tag{S17}$$

with $\mathbf{E}_L$ the electric field amplitude of the laser, and $\nu_L$ the laser frequency. $\overleftrightarrow{\alpha}_k$ is the Raman polarizability tensor, given by[7]

$$\overleftrightarrow{\alpha}_k = b_k \left(\frac{\partial \overleftrightarrow{\alpha}}{\partial Q_k}\right)_0, \tag{S18}$$

with the linear optical polarizability $\overleftrightarrow{\alpha}$. Gaussian DFT calculations provide the Raman activity[8]

$$\tilde{S}_k = 45{a'_k}^2 + 7{\gamma'_k}^2, \tag{S19}$$

with

$$a'_k = \frac{1}{3}(\alpha'_{k,xx} + \alpha'_{k,yy} + \alpha'_{k,zz}) \tag{S20}$$

and

$$\begin{aligned}{\gamma'_k}^2 = \frac{1}{2}\Big[&(\alpha'_{k,xx} - \alpha'_{k,yy})^2 + (\alpha'_{k,yy} - \alpha'_{k,zz})^2 + (\alpha'_{k,zz} - \alpha'_{k,xx})^2 \\ &+ 6({\alpha'_{k,xy}}^2 + {\alpha'_{k,yz}}^2 + {\alpha'_{k,zx}}^2)\Big],\end{aligned} \tag{S21}$$

where

$$\alpha'_{k,ij} = \left(\frac{\partial \alpha_{ij}}{\partial Q_k}\right)_0. \tag{S22}$$

The Raman activity from DFT (in $\text{Å}^4/\mathrm{amu}$) is converted to SI units ($\mathrm{C\ m\ kg^{-1/2}\ V^{-1}}$) by[8]

$$S_k = (4\pi\varepsilon_0)^2\, \tilde{S}_k. \tag{S23}$$

For a Raman tensor with dominant $zz$ component $S_k = 12{\alpha'_{k,zz}}^2$ the Raman polarizability is given by

$$\alpha_k = b_k\sqrt{S_k/12}. \quad \text{(S24)}$$

The electric field amplitude of a laser with intensity $I_L$ in W/m$^2$ is $E_L = \sqrt{2I_L/\varepsilon_0 c}$ and enhanced by a factor $EF$ in a plasmonic cavity. The induced Raman dipole is thus

$$p_k^{Raman} = \sqrt{\frac{\hbar S_k I_L}{24\pi\nu_k \varepsilon_0 c}}\, EF. \quad \text{(S25)}$$

Finally, Raman dipoles can be also induced by the vacuum electromagnetic field in the cavity, which has a field amplitude

$$E_{vac} = \sqrt{\frac{\hbar\pi\nu_L}{\varepsilon_0 V_m}}, \quad \text{(S26)}$$

where $V_m$ is the mode volume of the plasmonic cavity. This leads to an induced Raman dipole of

$$p_{k,vac}^{Raman} = \sqrt{\frac{\hbar^2\nu_L S_k}{48\varepsilon_0 \nu_k V_m}}. \quad \text{(S27)}$$

Table S1 lists the IR and Raman dipole moments for the $NO_2$ stretch vibration of 4NTP, which we calculated from DFT (Fig. S3). The induced Raman dipoles are one to three orders of magnitude smaller than the IR transition dipole moment.

**Table S1.** IR and Raman dipole moments of the $NO_2$ stretch vibration of 4NTP, estimated from DFT IR intensity $I_{\mathrm{IR}}$ and Raman activity $\tilde{S}$. The $E$ fields in NPoM gaps are calculated for a laser intensity of $10\ \mu\mathrm{W}/\mu\mathrm{m}^2$ and field enhancement $EF$ = 200 for direct excitation with a laser $E_L$, or a mode volume of $V_m = 150\ \mathrm{nm}^3$ for the vacuum electromagnetic field in the cavity $E_{vac}$ (see two columns). The frequency shifts are calculated for a dimer of parallel dipoles, separated by 5 Å, with dielectric function of the surrounding medium of $\varepsilon_m = 2.25$.

| | | E field (V/nm) | Dipole moment (D) | Frequency shift for dimer (cm$^{-1}$) |
|---|---|---|---|---|
| Induced Raman dipole | $\tilde{S} \simeq 1{,}200\,\frac{\text{Å}^4}{\mathrm{amu}}$ | $E_L \cdot EF = 0.039$<br>$E_{vac} = 0.34$ | 0.00066<br>0.013 | $7.7 \cdot 10^{-6}$<br>$3.0 \cdot 10^{-3}$ |
| IR transition dipole | $I_{\mathrm{IR}} = 690\ \mathrm{km/mol}$ | - | 0.45 | 3.6 |

## Section S4: DFT simulations

We calculated IR intensity and Raman activity of the four molecules 4NTP, MBN, TFTP and BPT using DFT (Fig. S3, see also Section S1). Each molecule was attached to a single Au atom, to account for the thiol bond and chemical changes to the molecule. Comparing the spectra of the four molecules it is apparent that the $NO_2$ vibration of 4NTP has the largest IR intensity and is also expected to be the strongest vibration in the Raman spectrum (Fig. S3a). The Raman activity of 4NTP is probably underestimated as the molecule has an electronic excitation close to 633 nm, and the resulting resonance Raman effect is not included in the Raman calculation algorithm in Gaussian.[1]

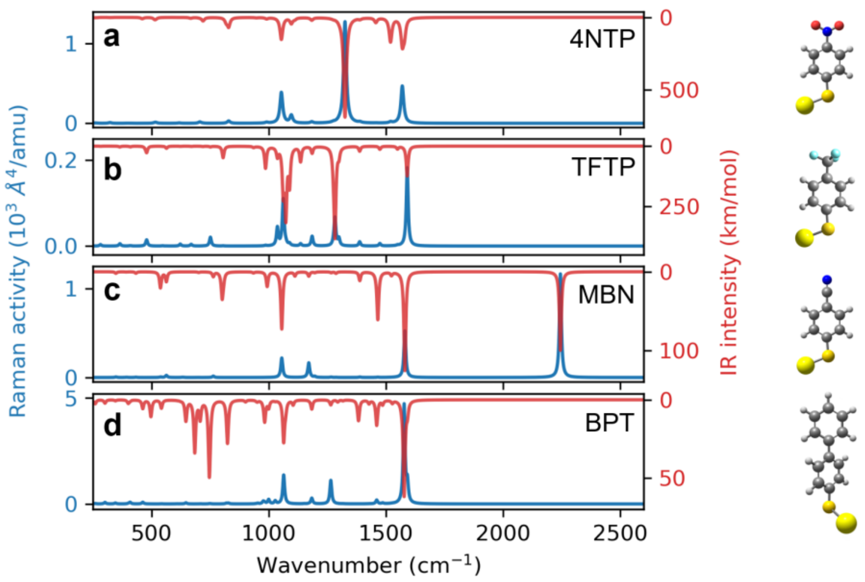


**Figure S3.** DFT calculation of the Raman activity (blue) and IR intensity (red) of the molecules (a) 4-nitrothiophenol (4NTP), (b) 4-(trifluoromethyl)-thiophenol (TFTP) (c) 4-mercaptobenzonitrile (MBN) and (d) biphenyl-4-thiol (BPT). All frequencies are scaled by a factor 0.965.

Furthermore, we calculated the collective vibrations of a 4NTP tetramer that is attached to Au atoms (Fig. S4). The gold-4NTP interface was modelled with one gold atom attached to each deprotonated thiol, with neutral total charge and singlet electronic state. For the geometry optimization we defined the nuclear positions in a Z-matrix coordinate system to keep the four molecules identical in a rectangular position, perpendicular to the surface (see Fig. S4g for geometry). Frequency calculations on the optimized structures were carried out in a Cartesian coordinate system, which allowed the construction of coupled vibrational modes. Figure S4a-d, top panels show the four collective vibrations of the $NO_2$ groups together with the frequency shifts $\Delta\nu_{\mathrm{DFT}}$ with respect to a single molecule. The vibrational frequency of the single molecule was obtained from a DFT calculation where three of the four molecules were frozen (see arrow in Fig. S4g). The frequency shifts and collective modes agree very well with the predictions of the microscopic theory (Fig. 4a-d, bottom panels). The collective mode in which all $NO_2$ groups vibrate in-phase is by far the dominant component in the Raman and IR spectra (Fig. S4e, f). The other collective vibrations lead to a peak asymmetry that is more pronounced for the Raman activity than the IR intensity.

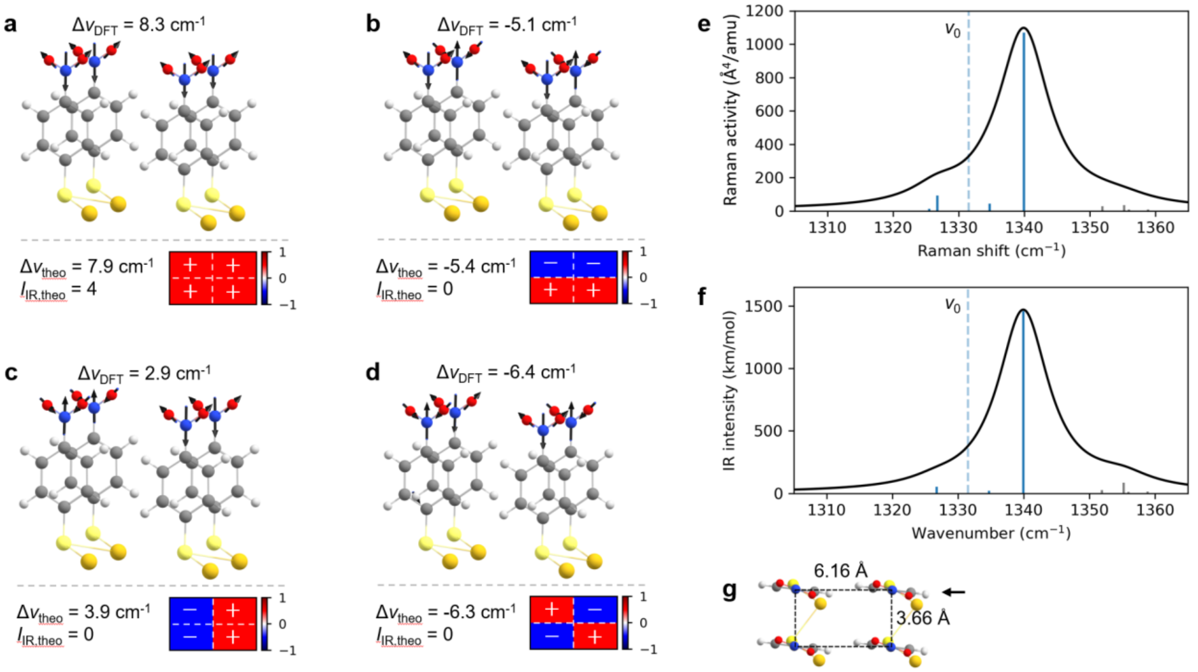


**Figure S4.** DFT calculation of the collective vibrations of a 4NTP tetramer. (a) – (d) Collective vibrations of the $NO_2$ head groups from DFT (top panels, black arrows) with labels giving the frequency shifts $\Delta\nu_{\rm DFT}$ with respect to uncoupled frequency $\nu_0$. Bottom panels show the frequency shifts $\Delta\nu_{\rm theo}$, IR activities $\left(\sum_j p_j\right)^2$ and collective modes predicted by the microscopic theory. (e) Raman activity and (f) IR intensity of the four collective vibrations (blue lines), compared to the uncoupled frequency $\nu_0$ (blue dashed line). The uncoupled frequency was obtained from a simulation of the tetramer, where three molecules were frozen. (g) Top view of the tetramer with labels giving dimensions. Arrow shows molecule that was used to obtain $\nu_0$ in a calculation where all other molecules where frozen. All vibrational frequencies were scaled by a factor 0.941.

## Section S5: Characterization of mixed SAMs with X-ray photoelectron spectroscopy

We characterized the pure and mixed self-assembled monolayers with X-ray photoelectron spectroscopy (XPS) and analyzed the spectra with the software CasaXPS. Figure S5 shows representative spectra of a pure SAM of 4NTP on an Au surface. The survey spectrum (Fig. S5a) is dominated by the peaks of Au. All other elements belong to the monolayer of 4NTP molecules, although some oxygen and carbon may be attributed to residual molecules in the vacuum chamber. Figure S5b shows the 4f peaks of Au, fit with a Shirley background and asymmetric Lorentzian LA(1.53, 243) peaks. We calibrated the binding energies in all spectra by shifting the Au $4f_{7/2}$ peak position to 83.95 eV. Figure S5c shows the N 1s peak, which belongs to the nitrogen atoms of the 4NTP molecules. The peak position 405.3 eV matches that of the nitro group.[9, 10] Other chemical bonds would lead to shifts of the N 1s peak by several eV, e.g. $-NH_2$ has a peak at 399 eV, see also Fig. S7a for -CN. Figure S5d shows the C 1s peak, which we fit with two peaks that are assigned to the aromatic backbone (284.2 eV) and terminal C atom bound to the nitro group (285.5 eV).[10] Figure S5e shows the S2p peak, which belongs to the thiol groups in all 4NTP molecules. The peak is well fit with a doublet split by 1.18 eV, an area ratio of 2 and the same FWHM for both components, as expected for an $2p_{1/2}$ - $2p_{3/2}$ doublet. The binding energies of 163.3 and 162.1 eV match those expected for bound thiols.[11] Unbound molecules would lead to another doublet at higher binding energies, which we did not detect.

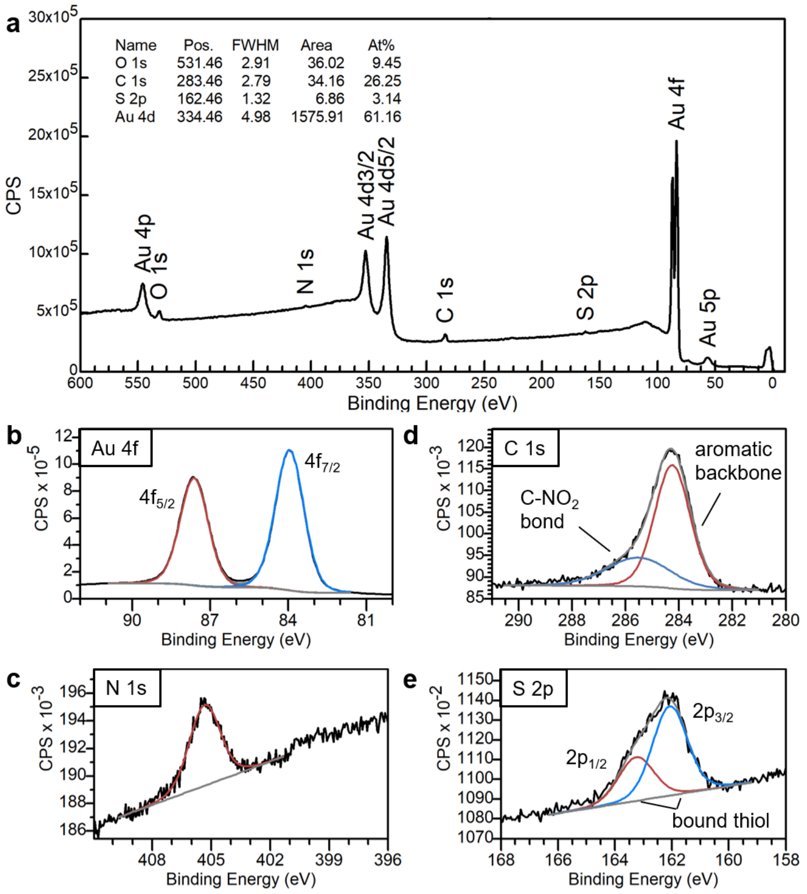


**Figure S5.** XPS spectra of a self-assembled monolayer of 4-nitrothiophenol on a 100 nm Au film evaporated on Si. (a) Survey spectrum with all relevant peaks labelled. Table gives elemental compositions estimated from peak areas. (b) Au 4f, (c) N 1s (d) C 1s and (e) S 2p peaks together with fits.

We estimated the packing density of 4NTP molecules from the S 2p to Au 4f peak ratio.[10] The inelastic mean free path of photoelectrons in Au is $\lambda_{Au} \approx 2.1$ nm, which corresponds to a sampling depth of $9 \pm 1$ Au layers.[12] The SAM of 4NTP molecules further attenuates the Au 4f photoelectrons by $e^{-d_c/\lambda_c} \approx 0.6$, with $d_c \approx 0.9$ nm the thickness of the SAM and $\lambda_c = 1.8$ nm the inelastic mean free path of photoelectrons in carbon.[12] The attenuation of the S2p photoelectrons is approximately $e^{-0.66\,\mathrm{nm}/\lambda_c} \approx 0.7$.[10] By accounting for the different attenuations of the S2p and Au 4f signals, the RSF factors $\mathrm{RSF}_{N1s} = 1.676$ and $\mathrm{RSF}_{S2p} = 1.881$ specific to instument used here, and assuming a (111) surface of an Au fcc lattice, we estimated a surface coverage of $0.35 \pm 0.04$ S atoms per primitive Au unit cell. This corresponds to a packing density of $\rho_{\mathrm{4NTP}} = (5.0 \pm 0.6) \cdot 10^{14}$ molecules/cm$^2$, or a lattice constant of $r_{\mathrm{4NTP}} = 4.8 \pm 0.3$ Å in a hexagonal lattice. Following similar steps, we also estimated the packing densities of BPT and MBN in pure SAMs (see Table S2). All uncertainties were estimated from the fits with CasaXPS, using a Monte Carlo Analysis.

**Table S2.** Packing densities and lattice constants of 4NTP, BPT and MBN in pure SAMs on a 100 nm evaporated Au film on Si, estimated from XPS S2p and Au4f peaks.

| | Packing density $\rho$ ($10^{14}$ molecules/cm$^2$) | Lattice constant $r$ (Å) |
|---|---|---|
| 4NTP | $5.0 \pm 0.6$ | $4.8 \pm 0.3$ |
| BPT | $4.4 \pm 0.5$ | $5.1 \pm 0.3$ |
| MBN | $6.0 \pm 0.7$ | $4.4 \pm 0.3$ |

Figure S6 shows the S2p and N1s spectra of mixed SAMs of 4NTP and BPT. The N1s peak intensity, belonging only to 4NTP molecules, decreases with increasing BPT fraction of the samples (Fig. S6b), while the S2p peak, belonging to all molecules, remains constant (Fig. S6a). We used the N1s/S2p peak ratios to measure the 4NTP fraction in the mixed SAMs (Fig. 1d). We normalized all peak ratios by the ratio N1s/S2p in a pure SAM of 4NTP to account for the different detection efficiency and attenuation of the N1s and S2p photoelectrons.

Figure S7a shows the XPS N1s spectra of mixed SAMs of 4NTP and MBN. The binding energies of N1s photoelectrons in -$NO_2$ (405.5 eV) belonging to 4NTP and -CN (398.5 eV) belonging to MBN differ from each other because of a chemical shift, which lets us distinguish the two molecules in the mixed SAMs. We used the XPS peak ratios $A_{N1s,4NTP}/(A_{N1s,4NTP} + A_{N1s,MBN})$ to measure the 4NTP fraction in the mixed SAMs (Fig. S7b). The mixing on the sample scales almost linearly with the mixing in solution during sample preparation. We fit the data with a Langmuir model of competitive adsorption[13]

$$\frac{\theta_{4NTP}}{\theta_{4NTP} + \theta_{MBN}} = \frac{x_{mol,4NTP} K}{1 + x_{mol,4NTP}(K-1)}, \tag{S28}$$

with $\theta_{4NTP}$, $\theta_{MBN}$ the molecular fractions on the sample and $x_{mol,4NTP}$ the molar 4NTP fraction in solution during sample preparation.

$$K = \frac{k_{ads}^{4NTP} k_d^{MBN}}{k_d^{4NTP} k_{ads}^{MBN}} \tag{S29}$$

is an equilibrium constant that accounts for the different adsorption rates $k_{ads}$ and desorption rates $k_{des}$ of the two molecules on the Au surface during SAM formation. A fit of the XPS peak ratios gives $K = 1.36$, which indicates a similar binding affinity of 4NTP and MBN to Au (Fig. S7b, solid line).

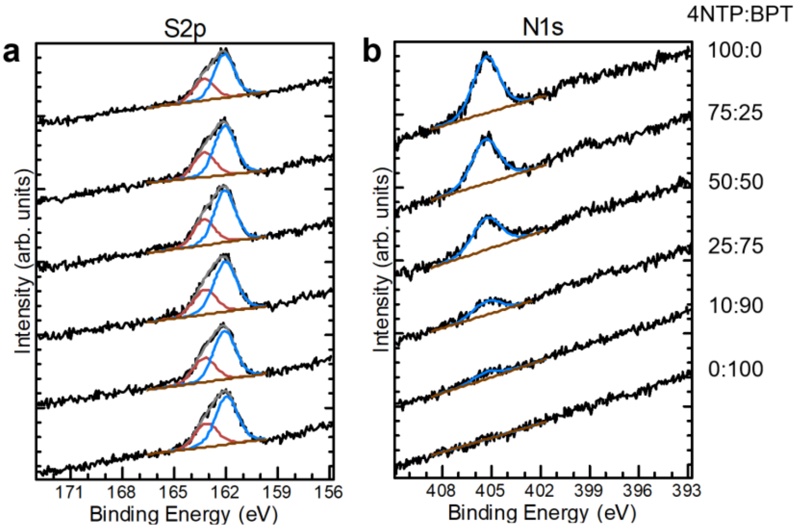


**Figure S6.** XPS spectra of mixed self-assembled monolayers of the molecules 4NTP and BPT. (a) S 2p peak, which originates from the thiol groups in all the molecules of the mixed SAM and (b) N 1s peak, which only belongs to the 4NTP molecules. Labels give mixing fraction during sample preparation.

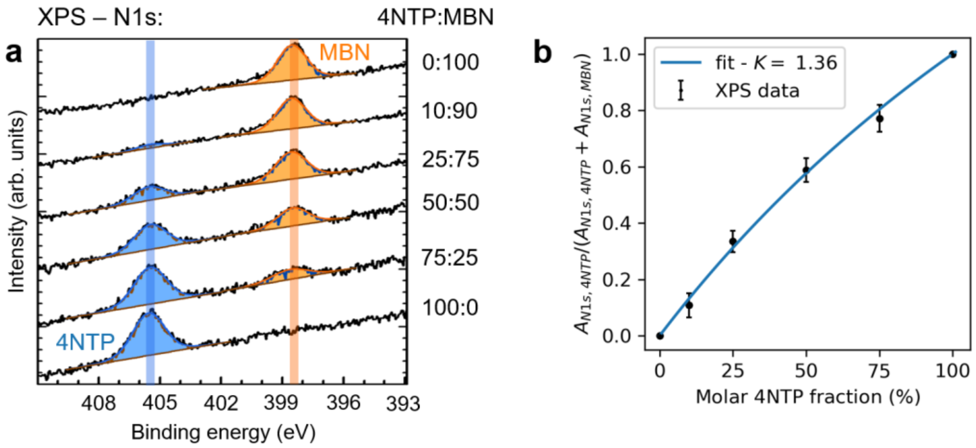


**Figure S7.** XPS spectra of mixed self-assembled monolayers of the molecules 4NTP and MBN. (a) N 1s peaks which belong to the nitro group of 4NTP (405.5 eV) and the nitrile group of MBN (398.5 eV). The different binding energies originate from a chemical shift. Labels give mixing fraction during sample preparation. (b) Mixing fraction from XPS peak ratios vs molar mixing fraction during sample preparation. Data are fit with a Langmuir model of competitive adsorption which indicates almost identical binding affinity of the two molecules.

## Section S6: Cooperative frequency shifts in mixed SAMs of different types of molecules

In the following we describe how we analysed the vibrational frequencies from all individual SERS spectra of mixed SAMs of 4NTP and BPT. We fit the $NO_2$ vibration of 4NTP with a split Gaussian peak, i.e. a single peak with two full-width at half-maxima (FWHM1 and FWHM2) to the left and right of the central frequency (Fig. S8a). We chose this peak shape to account for the peak asymmetry of the $NO_2$ vibration and extract the dominant frequency. The fits with a single asymmetric peak are most robust with respect to noise and allowed us to consistently analyse the $NO_2$ peak position in the spectra of mixed SAMs, where the $NO_2$ peak becomes symmetric with decreasing 4NTP fraction (Fig. 2a,b). The peak asymmetry from fits of all individual spectra is shown in Fig. S8c. Fitting the $NO_2$ peak, e.g., with two or more peaks would be impractical for the spectra of mixed SAMs and low 4NTP fraction.

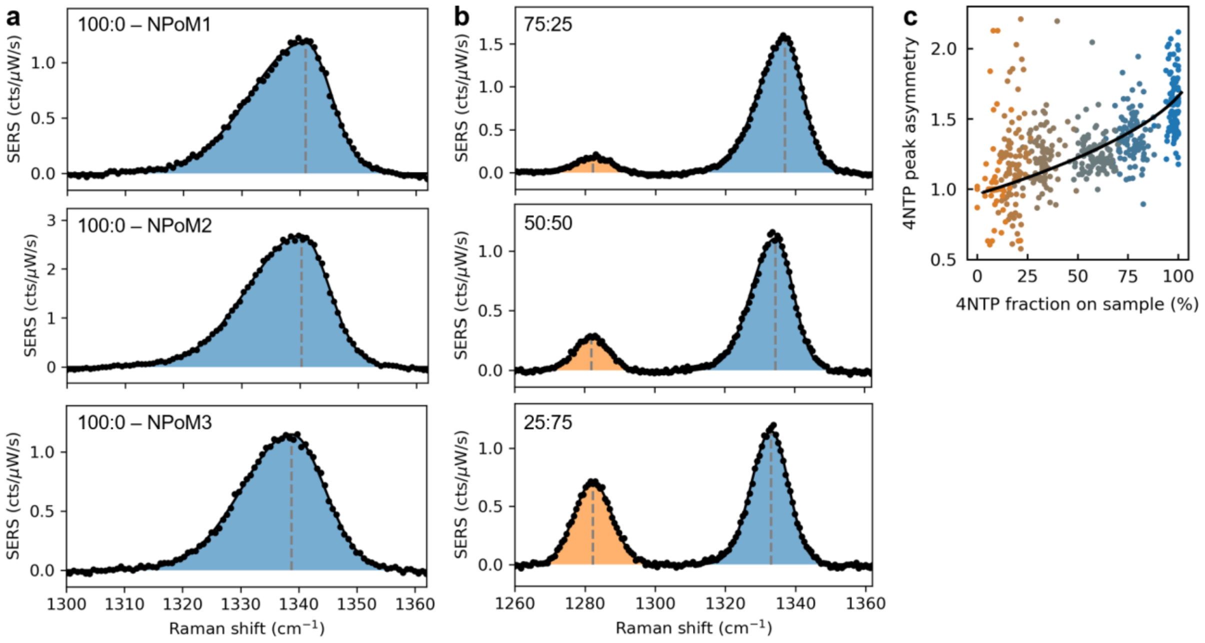


**Figure S8.** Analysis of SERS peak positions in mixed SAMs of 4NTP and BPT. (a) Fits of $NO_2$ peak of 4NTP with split Gaussian peak, for three representative SERS spectra recorded on individual NPoM cavities and pure SAMs of 4NTP. Dashed lines show $NO_2$ frequency $\nu_{\mathrm{NO2}}$. (b) Fits of ring-ring stretch vibration of BPT (orange) with Gaussian peak and $NO_2$ peak of 4NTP (blue) with split Gaussian peak for three different mixing fractions 4NTP:BPT, see labels. Panels show representative SERS spectra recorded on individual NPoM cavities. All spectra are background subtracted (linear slope). (c) Peak asymmetry (FWHM2/FWHM1) of $NO_2$ peak of 4NTP from fits of all individual spectra.

To better understand the peak asymmetry of the $NO_2$ vibration of 4NTP, we fit the SERS spectra of pure SAMs with several peak components and compare this to the prediction of the collective vibration theory (Fig. S9). We fix the peak components to the calculated frequencies of the three collective modes with the largest net dipoles (Fig. S9a and b: red, orange and blue). Furthermore, we add another component at the expected frequency for uncoupled molecules (grey). The $NO_2$ peak shape is well explained by the fits, even though we only use the amplitudes of the peak components as fit parameters (Fig. S9a). The analysis suggests that the highest-frequency collective mode, where all molecules vibrate in-phase, is by far the dominant collective vibration (Fig. S9a, red). Furthermore, there is a significant fraction of molecules that remain uncoupled, which is the major reason for the peak asymmetry (Fig. S9a, grey).

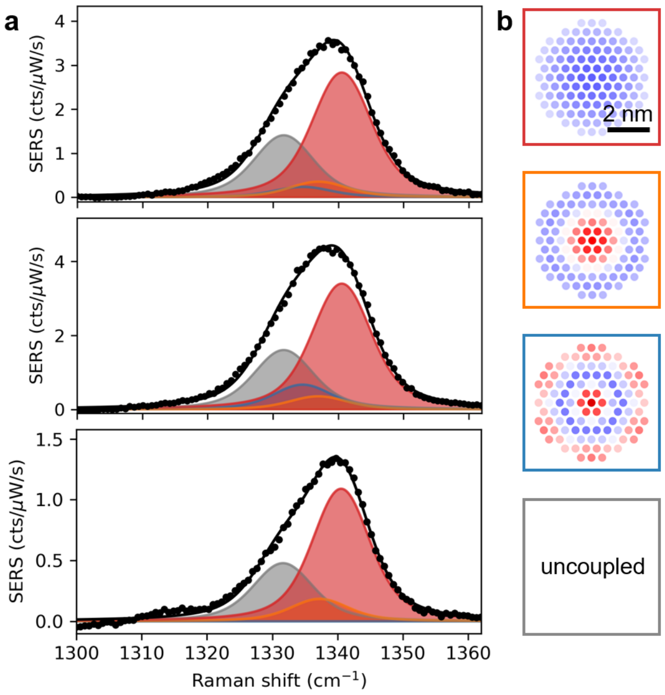


**Figure S9.** Analysis of $NO_2$ peak asymmetry in the SERS spectra of 4NTP with collective vibration model. (a) Representative fits of SERS spectra recorded on individual NPoM cavities. Spectra were fit with four Voigt peaks using peak positions expected from theory for the three collective modes with the largest net dipole moment $\Delta\nu_{NO2}$ = 8.5 cm$^{-1}$ (red), $\Delta\nu_{NO2}$ = 5.3 cm$^{-1}$ (orange), $\Delta\nu_{NO2}$ = 2.5 cm$^{-1}$ (blue) and uncoupled molecules $\Delta\nu_{NO2}$ = 0 cm$^{-1}$ (grey). The FWHMs were fixed to those obtained from a fit of the uncoupled $NO_2$ peak in a 5:95 mixed SAM, with frequency $\nu_{NO2}$ = 1331.8 cm$^{-1}$. Only peak amplitudes were used as fit parameters. All spectra are background subtracted (linear slope). (b) Eigenvectors of the three collective modes with the largest net dipole moment. Parameters: Same as in Fig. 2d, for hexagonal lattice with spherical domain shape and ~100 coupled dipoles.

We determined the mixing of 4NTP and BPT molecules on the sample from the SERS peak ratios of the ring vibration of 4NTP (1570 cm$^{-1}$) and the ring vibrations of BPT (1586 and 1600 cm$^{-1}$), see Fig. 1b-d in the main paper. In Figure S10a we show peak fits of the SERS average spectra for each mixing fraction, which are representative for our analysis. We evaluated the ring vibrations instead of the 1340 cm$^{-1}$ and 1280 cm$^{-1}$ vibrations as we were not sure whether intermolecular coupling affects the SERS intensity. Figure S10b shows a comparison with the peak ratios of the 1340 cm$^{-1}$ and 1280 cm$^{-1}$ vibrations. Both peak ratios show agreement on the fractional coverage estimated.

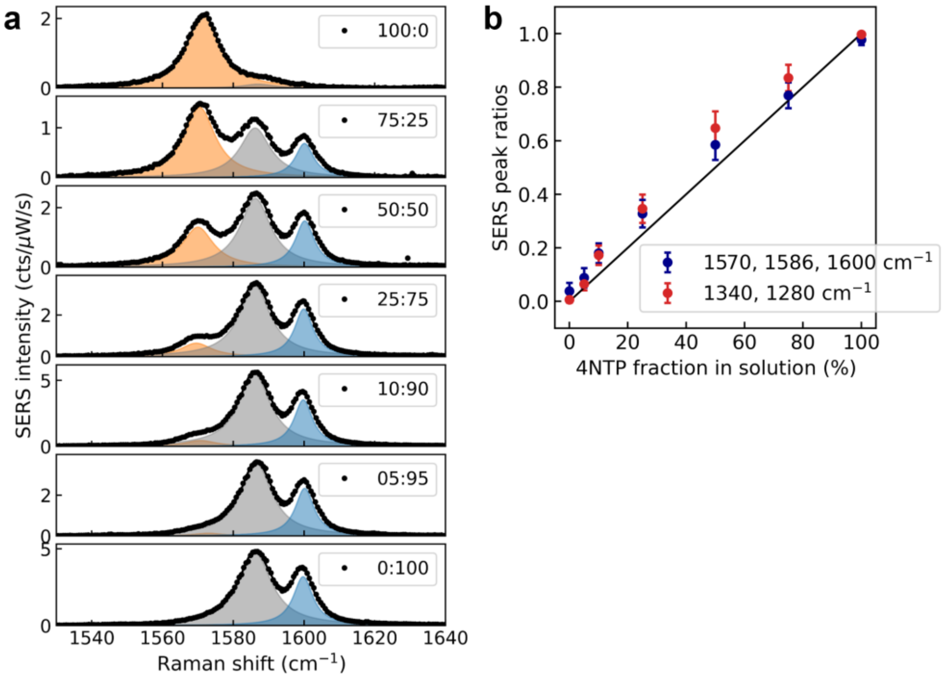


**Figure S10.** (a) Fit of ring vibrations in SERS average spectra using three Lorentzians to extract the peak components of 4NTP (1570 cm$^{-1}$, orange) and BPT (1586 cm$^{-1}$, grey and 1600 cm$^{-1}$, blue). (b) Comparison of SERS peak ratios from ring vibrations [$A_{4NTP}/(A_{4NTP} + 0.3A_{BPT})$, blue] with peak ratios from 1340 cm$^{-1}$ vibration of 4NTP and 1280 cm$^{-1}$ vibration of BPT [$A_{4NTP}/(A_{4NTP} + 2.9A_{BPT})$, red].

We also analysed the SERS spectra of mixed SAMs of the molecules 4NTP and 4-mercaptobenzonitrile (MBN) (Fig. S11). The vibrations of the $NO_2$ and CN head groups of both molecules have very different frequencies of $\nu_{\mathrm{NO2}}$ ≈ 1340 cm$^{-1}$ and $\nu_{\mathrm{CN}}$ ≈ 2230 cm$^{-1}$ (Fig. S11a, b), which prevents a collective vibration between the two molecules. The frequency shift of the $NO_2$ vibration 8.6 $\pm$ 1.5 cm$^{-1}$ is much larger than that of the CN group 2.0 $\pm$ 0.5 cm$^{-1}$, and is similar to that of 4NTP in mixed SAMs with BPT. The mixing on the sample was assumed to be the same as in solution during sample preparation – see analysis of XPS spectra in Fig. S7.

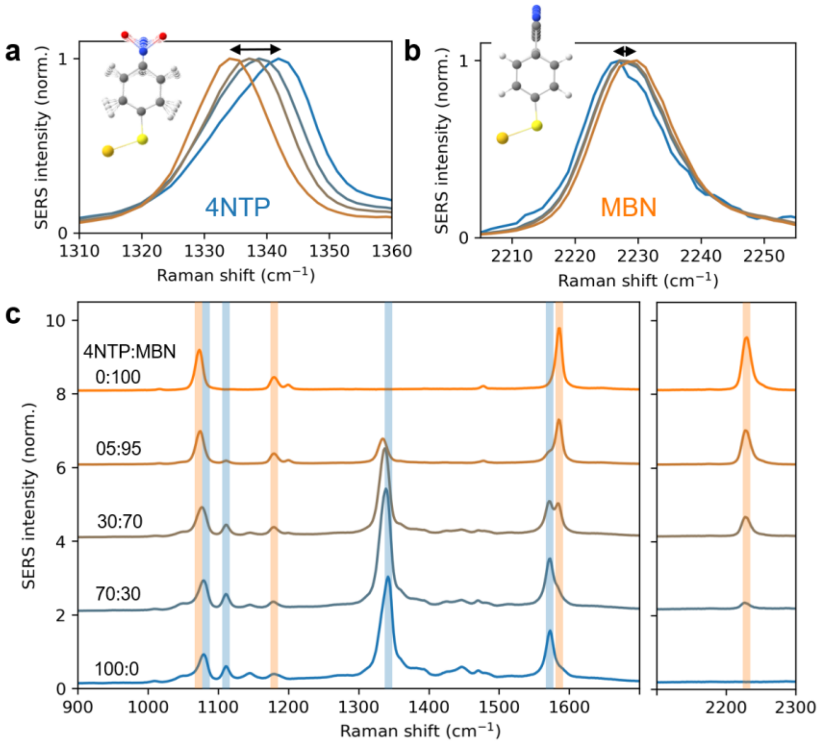


**Figure S11.** SERS average spectra of mixed SAMs of 4NTP (blue) and 4-mercaptobenzonitrile (MBN, orange). The spectra are calculated from SERS time traces of ~100 NPoM cavities for each mixing fraction. (a) $NO_2$ vibration of 4NTP, (b) CN vibration of MBN, and (c) full spectral range. Labels give molar mixing fraction during sample preparation. The spectra in (c) are normalized by the sum of peak areas of the ring vibrations of 4NTP (1570 cm$^{-1}$) and MBN (1586 cm$^{-1}$).

We also analysed the SERS spectra of mixed SAMs of the two molecules 4-(trifluoromethyl)-thiophenol (TFTP) and BPT (Fig. S12). For sample preparation, we mixed a 10 mM solution of TFTP with a 1 mM solution of BPT at ratios 50:50 and 20:80. We chose a much larger concentration of TFTP, as the two molecules have a different binding affinity to gold. The mixing on the sample is estimated as 50:50 and 10:90 from SERS peak ratios in the average spectra, using the $CF_3$ vibration of TFTP (1330 $cm^{-1}$) and the ring-ring stretch vibration of BPT (1280 $cm^{-1}$) (Fig. S12a, b). The frequency shift $5.8 \pm 1.1\ cm^{-1}$ of the $CF_3$ vibration is almost as large as that of the $NO_2$ vibration of 4NTP (Fig. S12c). The histograms in Fig. S12c indicate a good mixing of the two molecules on the sample.

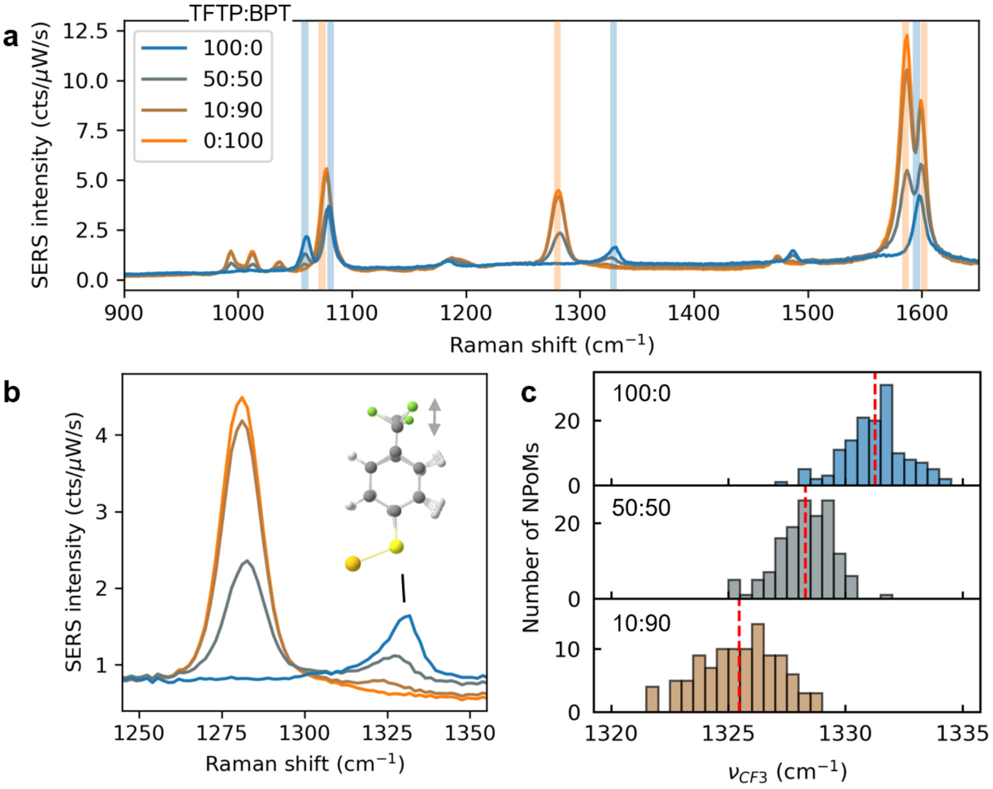


**Figure S12.** SERS characterization of mixed SAMs of 4-(trifluoromethyl)-thiophenol (TFTP, blue) and biphenyl-4-thiol (BPT, orange). (a) SERS average spectra calculated from SERS time traces of ~130 NPoM cavities for each mixing fraction. Labels give molar mixing fraction during sample preparation. (b) Spectral range of the TFTP $CF_3$ stretch vibration 1330 $cm^{-1}$ and the BPT ring-ring vibration at 1280 $cm^{-1}$. (c) Frequency of the $CF_3$ stretch vibration from fits of all individual spectra with a split Lorentzian peak, i.e., a single asymmetric peak with two FWHM to the left and right of the central frequency. Red dashed lines show the average frequencies for each mixing fraction.

We analyse the frequency shifts of several vibrations and molecules from the SERS spectra of mixed SAMs (Fig. S13). The vibrational frequencies are obtained from fits of the individual or average SERS spectra as detailed in the caption of Fig. S13. Only the $NO_2$ vibration of 4NTP and $CF_3$ vibration of TFTP show pronounced frequency shifts (Fig. S13a, d), while all other vibrations remain constant within 2 $cm^{-1}$. The frequency shifts between molecules in the pure SAMs and uncoupled molecules do not scale with the integrated SERS intensity (Fig. S13e).

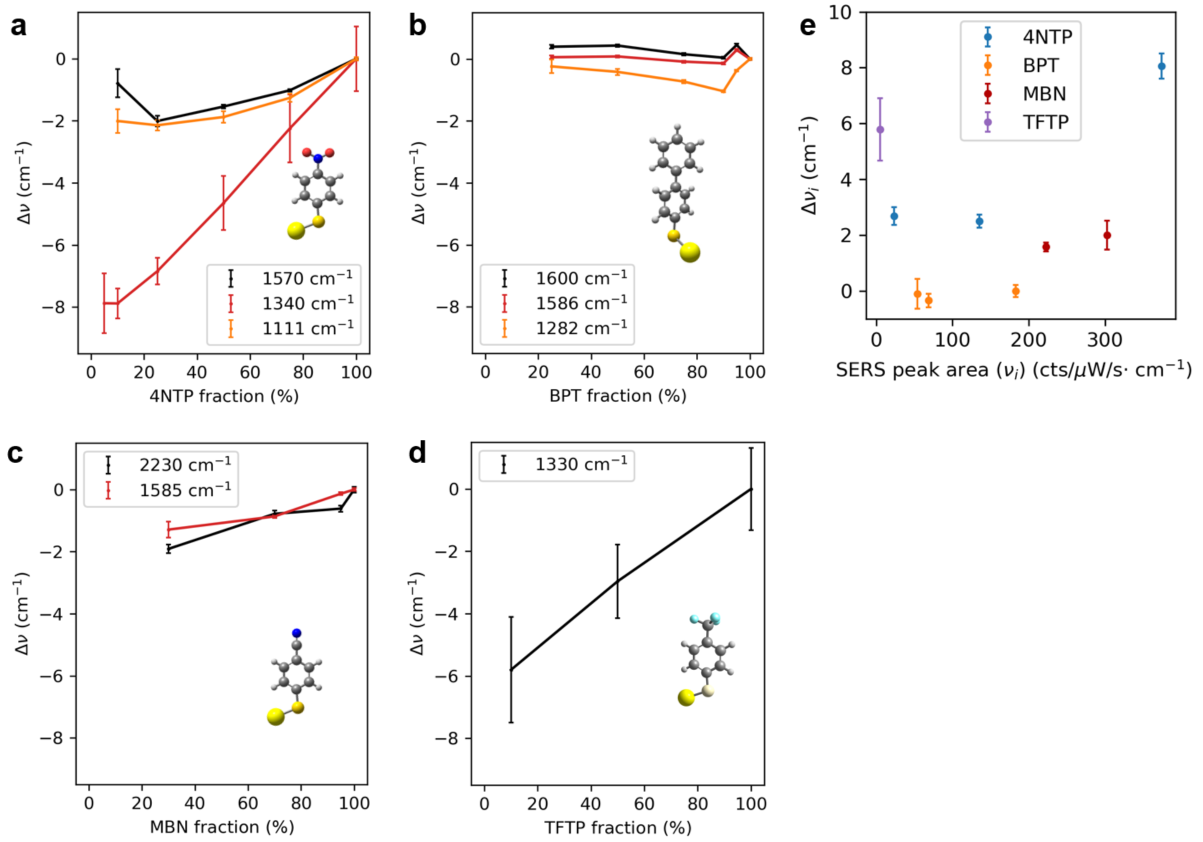


**Figure S13.** Vibrational frequency shifts $\Delta\nu$ of several molecules in mixed SAMs compared to pure SAM. (a) 4NTP in a mixed SAM with BPT. Data points for 1340 $cm^{-1}$ vibration from fits of individual spectra, while 1111 and 1570 $cm^{-1}$ are from fits of average spectra in Fig. 1b. (b) BPT in a mixed SAM with 4NTP. Frequency shifts from fits of average spectra in Fig. 1b. (c) MBN in a mixed SAM with 4NTP. Frequency shifts from fits of average spectra in Fig. S11. (d) TFTP in a mixed SAM with BPT. Frequency shifts from fits of individual spectra, see histograms in Fig. S12c. (e) Frequency shifts (between molecules in pure SAM and uncoupled molecules) *vs* SERS peak area of the pure SAMs (in cts/µW/s·$cm^{-1}$). Frequency shifts obtained from fits of data in (a-d) using $\Delta\nu(x_{mol}) = \Delta\nu \cdot x_{mol}^{3/2}$, where $x_{mol}$ is the fraction of the molecule in each sample.

## Section S7: Monitoring photochemical reactions with SERS

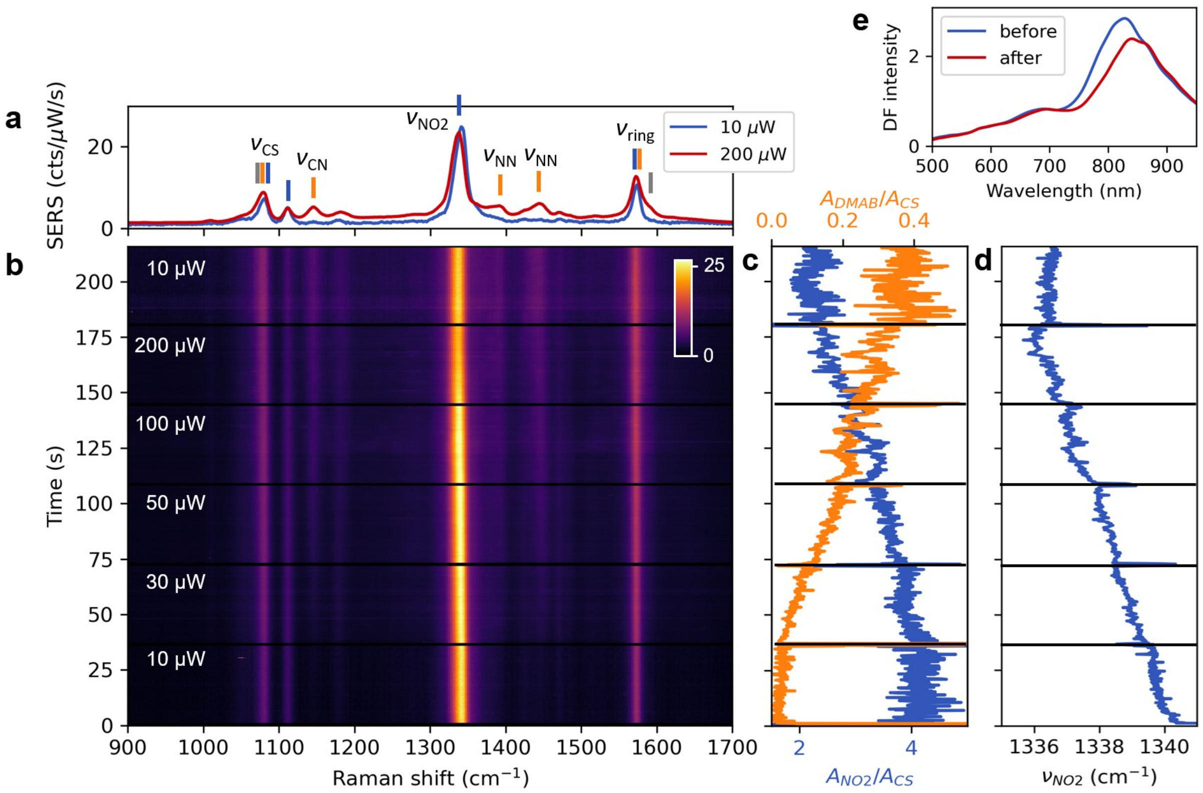


**Figure S14.** Photochemical reaction monitored with SERS on 45 NPoM cavities on a pure 4NTP SAM. All panels show average spectra of all NPoM cavities. (a) Time-averaged SERS spectra at 10 µW (blue) and 200 µW (red) from time trace in (b). Blue lines show expected vibrations of 4NTP, orange of DMAB, and gray of 4-amino thiophenol. (b) Average SERS time trace while stepwise increasing the laser power (see labels). Color scale bar in cts/µW/s. (c) SERS intensity ratio of $NO_2$ vibration of 4NTP (blue) and ring-sulphur vibration (1080 cm$^{-1}$, belonging to all molecules), and DMAB (1435 cm$^{-1}$, orange) from fit of spectra in (b). (d) Frequency of $NO_2$ vibration, from fit of spectra in (b). (e) Dark field (DF) average spectrum before (blue) and after (red) recording the SERS spectra.

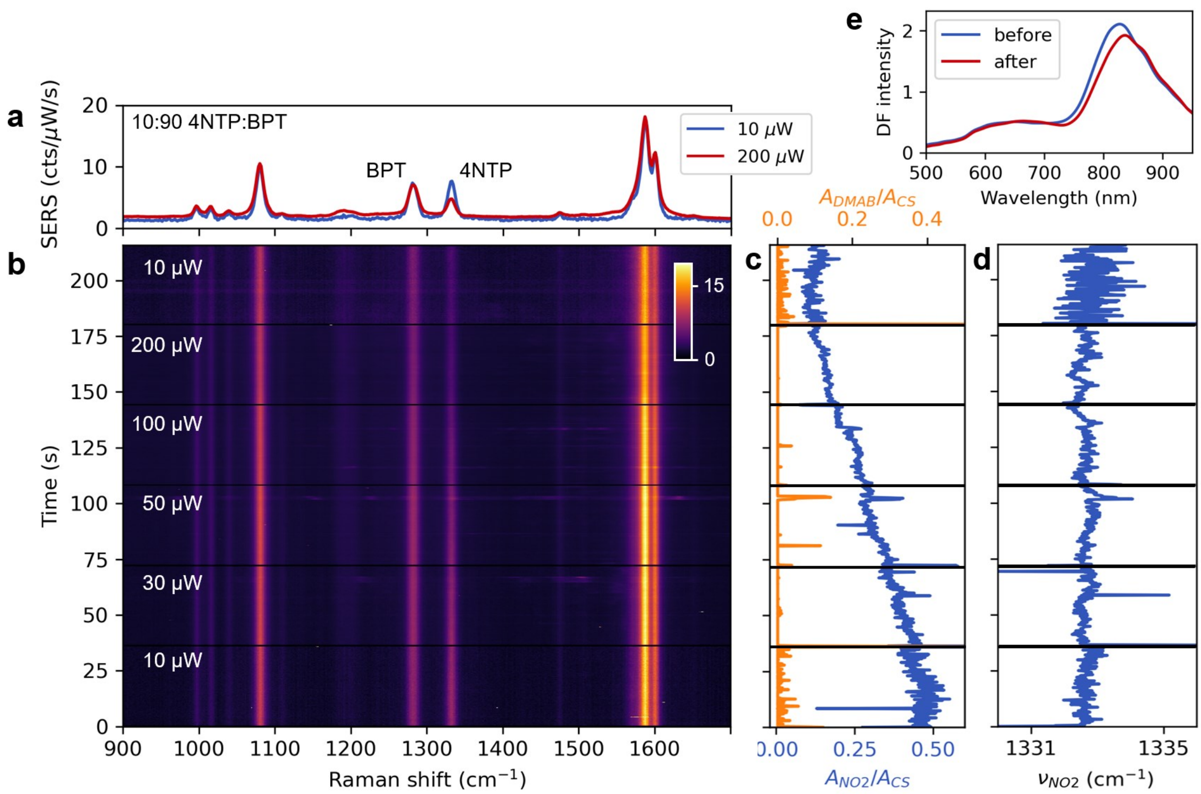


**Figure S15.** Photochemical reaction monitored with SERS on 21 NPoM cavities on a 10:90 mixed SAM of 4NTP and BPT. All panels show average spectra from all NPoM cavities. (a) Time-averaged SERS spectra at 10 µW (blue) and 200 µW (red) from time trace in (b). Complementary vibrations of BPT and 4NTP are labelled. (b) Average SERS time trace while stepwise increasing the laser power (see labels). Color scale bar in cts/µW/s. (c) SERS intensity ratio of $NO_2$ vibration of 4NTP (blue) and ring-sulphur vibration (1080 cm$^{-1}$, belonging to all molecules), and DMAB (1435 cm$^{-1}$, orange) from fit of spectra in (b). No DMAB peak is detectable. (d) Frequency of $NO_2$ vibration, from fit of spectra in (b). (e) Dark field (DF) average spectrum before (blue) and after (red) recording the SERS spectra.

## Section S8: Scanning tunnelling microscopy of molecular monolayer

We characterized a SAM of BPT molecules on a template stripped Au surface with scanning tunnelling microscopy (STM), see Fig. S16 and Section S1. Image analysis was performed using Gwyddion SPM analysis software. All images underwent a plane levelling procedure and a line-wise offset correction. To highlight the smaller scale 2D structure arising from SAM formation the larger scale surface features associated with the template stripped Au surface were removed using a median filter to perform a background subtraction. 2D Fourier transforms (2D-FFT) were then produced using the background subtracted images. The 2D-FFT's were filtered to highlight the structure resulting from the SAM (see example STM images shown in Figure S16). 2D unit mesh parameters for the SAM structures were measured from the Fourier filtered images. This process was repeated for at least 10 different images collected at different locations on the sample surface to produce average values for the unit mesh parameters. Error values in the unit mesh parameters are given as one standard deviation of the repeat measurements.

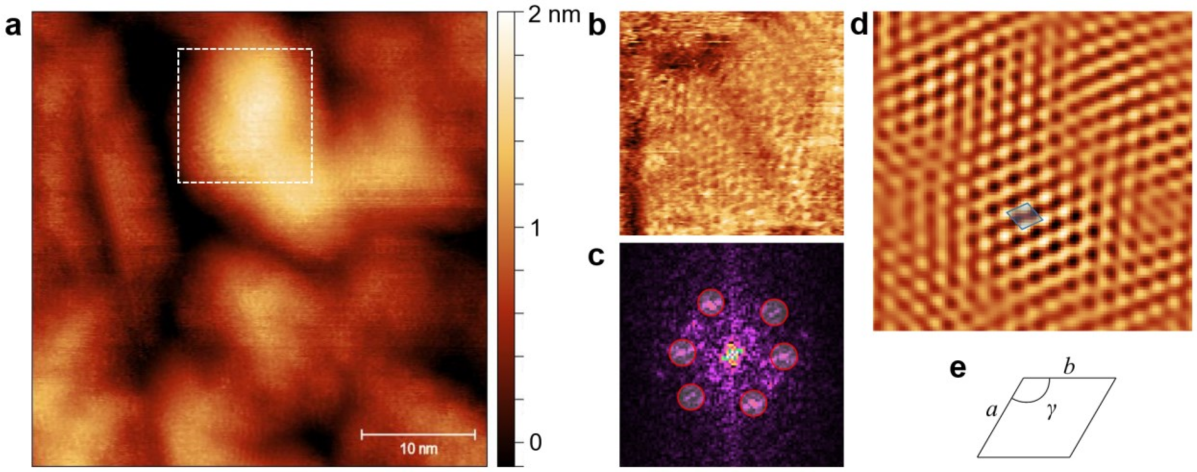


f

| $a$ / Å | $b$ / Å | $\gamma$ / ° |
|---|---|---|
| 5.5 ± 0.4 | 5.5 ± 0.4 | 119 ± 5 |

**Figure S16.** STM analysis of a BPT SAM on template stripped Au. (a) STM image, before background removal and Fourier filtering, showing the larger scale structure of the template stripped Au surface. Image size 50 nm, scanning parameters $I_t$ = 0.3 nA and $V_t$ = +0.3 V. (b) STM after background removal via median filter; Image size 15 nm, scanning parameters $I_t$ = 0.3 nA and $V_t$ = +0.3 V. (c) 2D-FFT of the STM image shown in (b). The red circles in (c) show the regions of the 2D-FFT that have been used to generate the Fourier filtered image shown in (d). (e) shows a schematic of the 2D unit mesh for the SAM structure with unit mesh parameters $a$, $b$ and angle $\gamma$. (f) Average values for the unit mesh parameters of the BPT SAM calculated from 10 repeat measurements taken from different images. The quoted error values are one standard deviation of the repeat measurements.

REFERENCES


(1) Juergensen, S.; Kusch, P.; Reich, S. Resonant Raman Scattering of 4-Nitrothiophenol. *physica status solidi (b)* **2020**, *257* (12), 2000295. DOI: 10.1002/pssb.202000295.

(2) Hexter, R. M. Intermolecular Coupling of Vibrations in Molecular Crystals: A Vibrational Exciton Approach. *The Journal of Chemical Physics* **1960**, *33* (6), 1833-1841. DOI: 10.1063/1.1731514.

(3) Muller Eric, A.; Gray Thomas, P.; Zhou, Z.; Cheng, X.; Khatib, O.; Bechtel Hans, A.; Raschke Markus, B. Vibrational exciton nanoimaging of phases and domains in porphyrin nanocrystals. *Proceedings of the National Academy of Sciences* **2020**, *117* (13), 7030-7037. DOI: 10.1073/pnas.1914172117.

(4) Gray, T. P.; Nishida, J.; Johnson, S. C.; Raschke, M. B. 2D Vibrational Exciton Nanoimaging of Domain Formation in Self-Assembled Monolayers. *Nano Letters* **2021**, *21* (13), 5754-5759. DOI: 10.1021/acs.nanolett.1c01515.

(5) Jakob, L. A.; Deacon, W. M.; Zhang, Y.; de Nijs, B.; Pavlenko, E.; Hu, S.; Carnegie, C.; Neuman, T.; Esteban, R.; Aizpurua, J.; et al. Softening molecular bonds through the giant optomechanical spring effect in plasmonic nanocavities. arXiv:2204.09641, 2022.

(6) Olmon, R. L.; Slovick, B.; Johnson, T. W.; Shelton, D.; Oh, S.-H.; Boreman, G. D.; Raschke, M. B. Optical dielectric function of gold. *Physical Review B* **2012**, *86* (23), 235147. DOI: 10.1103/PhysRevB.86.235147.

(7) Neugebauer, J.; Reiher, M.; Kind, C.; Hess, B. A. Quantum chemical calculation of vibrational spectra of large molecules—Raman and IR spectra for Buckminsterfullerene. *Journal of Computational Chemistry* **2002**, *23* (9), 895-910. DOI: 10.1002/jcc.10089.

(8) Le Ru, E. C.; Etchegoin, P. G. *Principles of surface-enhanced Raman spectroscopy : and related plasmonic effects*; Amsterdam ; Boston : Elsevier, 2009.

(9) Nielsen, J. U.; Esplandiu, M. J.; Kolb, D. M. 4-Nitrothiophenol SAM on Au(111) Investigated by in Situ STM, Electrochemistry, and XPS. *Langmuir* **2001**, *17* (11), 3454-3459. DOI: 10.1021/la001775o.

(10) Waske, P.; Wächter, T.; Terfort, A.; Zharnikov, M. Nitro-Substituted Aromatic Thiolate Self-Assembled Monolayers: Structural Properties and Electron Transfer upon Resonant Excitation of the Tail Group. *The Journal of Physical Chemistry C* **2014**, *118* (45), 26049-26060. DOI: 10.1021/jp507265k.

(11) Castner, D. G.; Hinds, K.; Grainger, D. W. X-ray Photoelectron Spectroscopy Sulfur 2p Study of Organic Thiol and Disulfide Binding Interactions with Gold Surfaces. *Langmuir* **1996**, *12* (21), 5083-5086. DOI: 10.1021/la960465w.

(12) Seah, M. P.; Dench, W. A. Quantitative electron spectroscopy of surfaces: A standard data base for electron inelastic mean free paths in solids. *Surface and Interface Analysis* **1979**, *1* (1), 2-11. DOI: 10.1002/sia.740010103.

(13) Stewart, A.; Zheng, S.; McCourt, M. R.; Bell, S. E. J. Controlling Assembly of Mixed Thiol Monolayers on Silver Nanoparticles to Tune Their Surface Properties. *ACS Nano* **2012**, *6* (5), 3718-3726. DOI: 10.1021/nn300629z.
